# An Interaction between RRP6 and SU(VAR)3-9 Targets RRP6 to Heterochromatin and Contributes to Heterochromatin Maintenance in *Drosophila melanogaster*

**DOI:** 10.1371/journal.pgen.1005523

**Published:** 2015-09-21

**Authors:** Andrea B. Eberle, Antonio Jordán-Pla, Antoni Gañez-Zapater, Viktoria Hessle, Gilad Silberberg, Anne von Euler, Rebecca A. Silverstein, Neus Visa

**Affiliations:** Department of Molecular Biosciences, The Wenner-Gren Institute, Stockholm University, Stockholm, Sweden; University of Cambridge, UNITED KINGDOM

## Abstract

RNA surveillance factors are involved in heterochromatin regulation in yeast and plants, but less is known about the possible roles of ribonucleases in the heterochromatin of animal cells. Here we show that RRP6, one of the catalytic subunits of the exosome, is necessary for silencing heterochromatic repeats in the genome of *Drosophila melanogaster*. We show that a fraction of RRP6 is associated with heterochromatin, and the analysis of the RRP6 interaction network revealed physical links between RRP6 and the heterochromatin factors HP1a, SU(VAR)3-9 and RPD3. Moreover, genome-wide studies of RRP6 occupancy in cells depleted of SU(VAR)3-9 demonstrated that SU(VAR)3-9 contributes to the tethering of RRP6 to a subset of heterochromatic loci. Depletion of the exosome ribonucleases RRP6 and DIS3 stabilizes heterochromatic transcripts derived from transposons and repetitive sequences, and renders the heterochromatin less compact, as shown by micrococcal nuclease and proximity-ligation assays. Such depletion also increases the amount of HP1a bound to heterochromatic transcripts. Taken together, our results suggest that SU(VAR)3-9 targets RRP6 to a subset of heterochromatic loci where RRP6 degrades chromatin-associated non-coding RNAs in a process that is necessary to maintain the packaging of the heterochromatin.

## Author Summary

Cells regulate the packaging of DNA in the chromatin, and an important process in the development of any eukaryote is the definition of chromatin states. Heterochromatin is a condensed form of chromatin that is usually silent. Short non-coding RNAs participate in the silencing of transposons in animal germ cells and in the establishment of heterochromatin states during early development. These non-coding RNAs guide histone methyltransferases to the histones in the chromatin, which in turn creates binding sites for other factors that keep the heterochromatin condensed. The non-coding RNAs that participate in the establishment of heterochromatic domains are active in germ cells and gonads, and other mechanisms must exist in animal somatic tissues to maintain the established patterns of heterochromatin throughout development. Here, we identify RRP6 as a protein that is necessary for maintaining the condensed state of heterochromatin in a subset of heterochromatic loci in the somatic fruitfly genome. RRP6 is a ribonuclease that plays many roles in RNA processing and in quality control of gene expression. We show that RRP6 is tethered to heterochromatin through an interaction with a histone methyltransferase, and that in the heterochromatin RRP6 acts on transcripts derived from repetitive sequences that need to be degraded to maintain the packaging of the heterochromatin. The importance of ribonucleases for the structure of the heterochromatin had been shown in yeast cells. Our findings show that RNA degradation participates in chromatin silencing also in animal cells.

## Introduction

Approximately 30% of the genome of *Drosophila melanogaster* is heterochromatic and is made up of transposons, transposon fragments and repetitive sequences with different degrees of complexity [1]. The heterochromatin contains high levels of heterochromatin-specific proteins, such as Heterochromatin Protein 1a (HP1a), and is enriched in certain patterns of post-translational modifications of the histone tails [2], [3]. Heterochromatin formation involves a cascade of histone modifications that are targeted to specific regions of the genome by complex protein-protein and protein-nucleic acid interactions. In the switch from euchromatin to heterochromatin, acetylated H3K9 (H3K9ac) is deacetylated by histone deacetylases such as RPD3/HDAC1. H3K9 is subsequently methylated by histone methyltransferases, and the methylated H3K9 (H3K9me) acts as a binding site for HP1a [3], [4]. The properties of the heterochromatin can spread along the chromatin fiber, and HP1a plays a central role in this process. The ability of HP1a to dimerize, to interact with the methyltransferase SU(VAR)3-9, and to bind H3K9me provides the basis for the spreading of heterochromatin [5]. An additional level of complexity in the establishment of heterochromatic states is provided by the fact that HP1a can also bind RNA in both *D*. *melanogaster* [6] and *Schizosaccharomyces pombe* [7]. Recent studies on Swi6, the HP1a ortholog of *S*. *pombe*, have shown that the interaction of Swi6 with RNA interferes with the binding of Swi6 with H3K9me [7].

Small non-coding RNAs are essential components of the regulation of chromatin packaging in different organisms [8]. Fission yeast uses siRNAs to silence heterochromatic sequences through the recruitment of the H3K9 methyltransferase Clr4 [4], [9]. RNAi-dependent mechanisms of heterochromatin assembly exist also in plants, where siRNAs direct *de novo* DNA methyltransferases to specific genomic sequences (reviewed in [10]). Animal cells use instead the piRNA pathway to trigger heterochromatin assembly and transposon silencing in the germ line. In *D*. *melanogaster*, non-coding RNAs transcribed from transposon-rich regions are processed into piRNAs, and a “Piwi-piRNA guidance hypothesis” has been recently proposed for the recruitment of SU(VAR)3-9 and HP1a to heterochromatin [11], [12], [13], [14]. The Piwi-piRNA system is active during early development and it directs the initial establishment of heterochromatin states not only in the germ line but also in somatic cells. Recent studies suggest that after embryogenesis, the patterns of heterochromatin packaging are maintained through cell divisions via piRNA-independent mechanisms [15], [16].

An important player in the regulation of non-coding RNAs is the exosome, a multiprotein complex with ribonucleolytic activity [17], [18], [19]. In *D*. *melanogaster*, the core of the exosome associates with two catalytic active subunits, RRP6 and DIS3 [20]. In the cell nucleus, the exosome is involved in the processing of many non-coding RNAs, including pre-rRNAs, and in the quality control of mRNA biogenesis [21], [22]. The exosome ribonucleases also degrade a large variety of unstable, non-coding RNAs in various organisms including *S*. *cerevisiae* [23], plants [24], and animals [25], [26]. Moreover, recent studies have revealed that RRP6 participates in the regulation of enhancer RNAs [27] and in the degradation of unstable transcripts synthesized at DNA double-strand breaks [28].

The exosome has been functionally linked to the methylation of H3K9 in heterochromatin [29]. In *S*. *pombe*, RRP6 participates in the assembly of centromeric heterochromatin through an RNAi-independent mechanism [30], and collaborates with the RNAi machinery to silence developmentally regulated loci and retrotransposons [31]. Much less is known about the possible links between RRP6 and heterochromatin in animals. We have observed that a fraction of RRP6 is associated with heterochromatin in the genome of *D*. *melanogaster*, and we have identified physical interactions between RRP6 and several heterochromatin factors, including HP1a, SU(VAR)3-9, and RPD3. Our results show that SU(VAR)3-9 promotes the targeting of RRP6 to transposon-rich heterochromatic loci. In these loci, RRP6 contributes to maintaining the structure of the heterochromatin by degrading non-coding RNAs that would otherwise compromise the packaging of the chromatin.

## Results

### RRP6 is associated with heterochromatic regions in the genome of *D*. *melanogaster*

**Fig 1:**
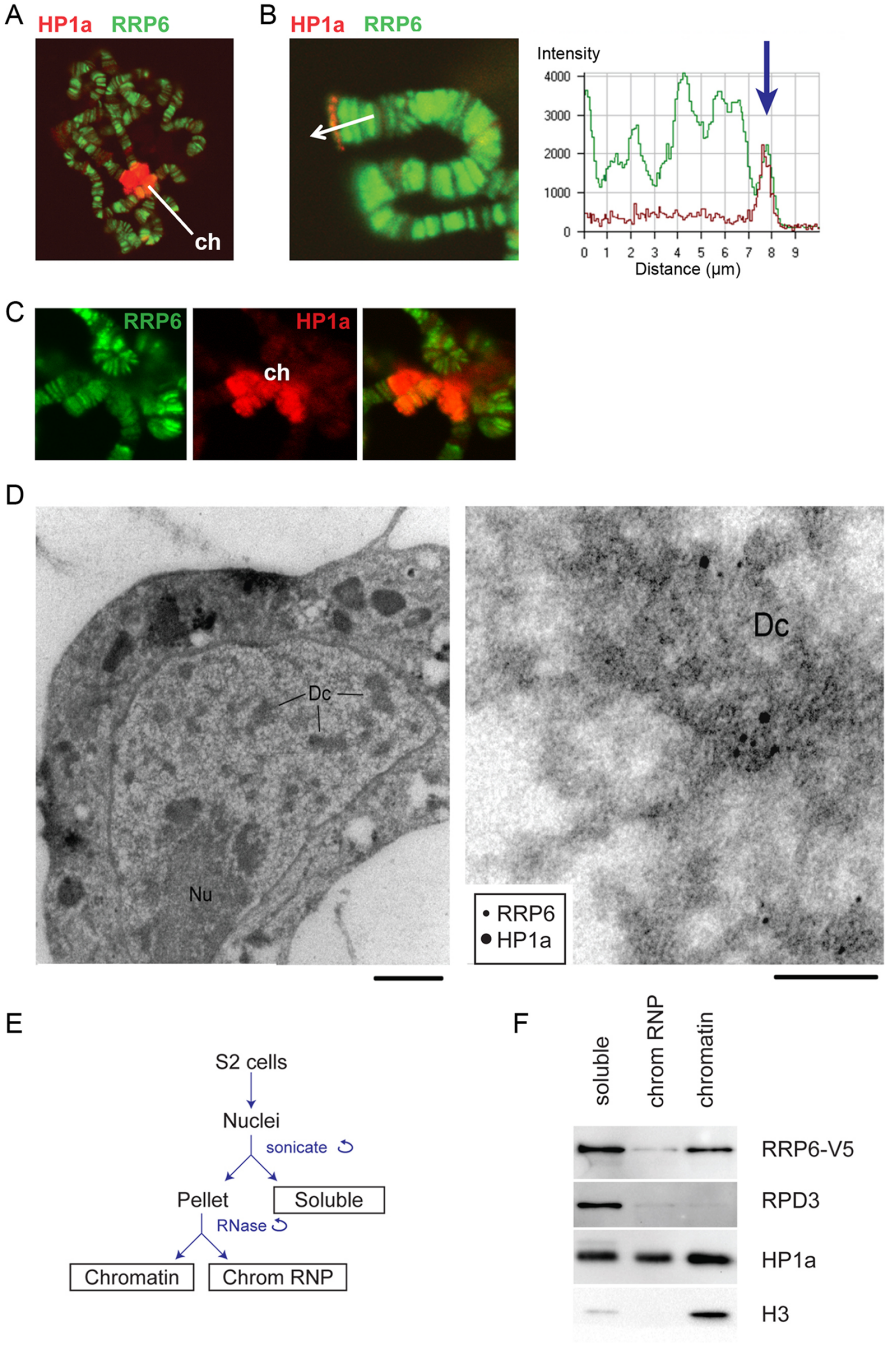
RRP6 is associated with heterochromatin *in vivo*. (A) Salivary gland polytene chromosomes immunostained with antibodies against RRP6 (green) and HP1a (red). The figure shows an overview micrograph. ch: chromocenter. (B) A detail showing a telomere stained with antibodies against RRP6 and HP1a, as in A. The fluorescence profile in the right part of the image shows the co-variation of both signals along the telomeric region. (C) A detail showing the chromocenter (ch) stained with antibodies against RRP6 and HP1a, as in A. (D) Co-localization of HP1a and RRP6 in dense chromatin in S2 cells analyzed by immuno-EM. An overview of a thin section through the nucleus of a cell is shown to the left. The bar represents 1 μm. A high-magnification micrograph shows co-localization of HP1a (12 nm gold) and RRP6 (6 nm gold) in the dense chromatin (*Dc*). The bar represents 100 nm. (E) The fractionation scheme used to isolate the different nuclear fractions in S2 cells: soluble (nucleoplasm), chromosomal RNP, and chromatin. (F) The distribution of HP1a, RPD3, and RRP6-V5 in the different nuclear fractions in S2 cells analyzed by Western blotting. The experiment was carried out in cells that expressed V5-tagged RRP6. Histone H3 was used as a control.

We analyzed the localization of RRP6 in salivary gland polytene chromosomes by immunofluorescence (IF), and we used an antibody against HP1a as a marker for heterochromatin. RRP6 was associated with many bands throughout the chromosomes, but was also present in heterochromatin (Fig 1A–1C, [32]). Although the overall distributions of HP1a and RRP6 were very different from each other, both proteins overlapped in heterochromatic regions, for example at telomeres (Fig 1B) and chromocenter (Fig 1C).

We also analyzed the association of RRP6 with heterochromatin in S2 cells by IF (S1 Fig) and by immuno-electron microscopy (IEM, Fig 1D). The IEM analysis revealed that RRP6 and HP1a were located in close proximity in dense chromatin areas (Fig 1D).

In another series of experiments, we used S2 cells that expressed V5-tagged RRP6 (S2-RRP6-V5 cells) and we analyzed RRP6-V5 under low-induction conditions to avoid overexpression artefacts. We applied a cell fractionation scheme previously established by Tyagi et al. [33], and the proteins in each of the different nuclear fractions (*soluble*, *chromosomal RNP* and *chromatin*) were analyzed by Western blotting. We observed that a fraction of RRP6 is associated with chromatin (Fig 1E and 1F). In these experiments, the *chromatin* fraction was digested with RNase A before centrifugation, which suggests that the binding of RRP6 to the *chromatin* fraction is not mediated by RNA.

### RRP6 interacts with RPD3, SU(VAR)3-9 and HP1a

**Fig 2:**
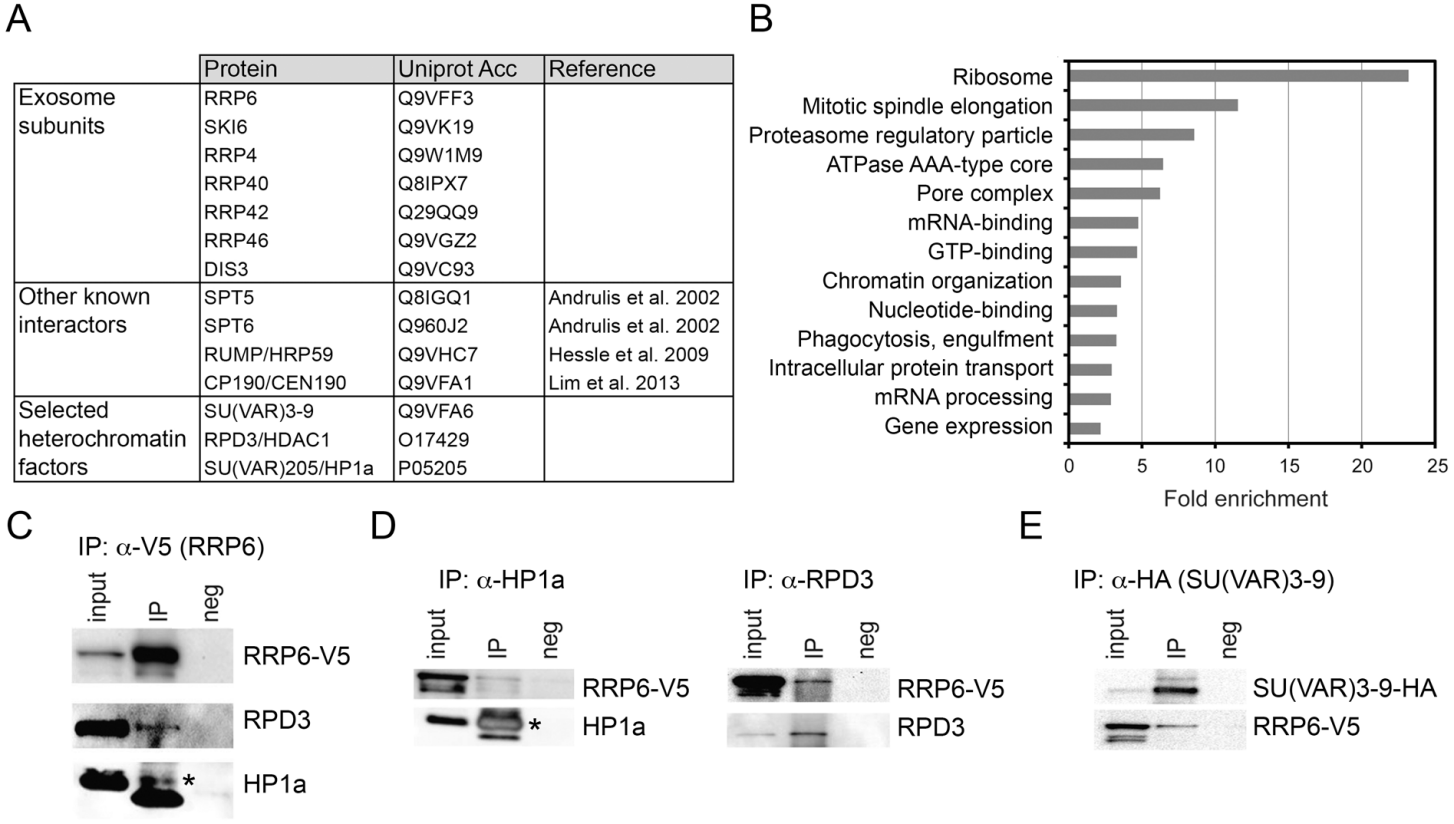
LC/MS-MS revealed interactions between the exosome and heterochromatin factors. (A) Some of the proteins co-immunoprecipitated with RRP6-V5 in S2 cells. The complete list of interactors is provided in S1 Table. (B) Enrichment of GO terms corresponding to RRP6 interactors. (C) Co-immunoprecipitation experiment performed with nuclear extracts of S2 cells expressing V5-tagged RRP6 using the anti-V5 antibody. The starting material (input), the immunoprecipitated proteins (IP) and the negative control were separated by SDS-PAGE and analysed by Western blotting using antibodies against V5, RPD3, and HP1a, as indicated. The band corresponding to HP1a is indicated with an asterisk. The thick band below HP1a is the light chain of the anti-V5 antibody (apparent molecular mass approx. 20 kDa). (D) Reciprocal co-immunoprecipitation experiments were performed in the RRP6-V5 cells using endogenous antibodies against HP1a and RPD3, as indicated. The asterisk indicates HP1a. (E) Co-immunoprecipitation experiment conducted in S2 cells that expressed simultaneously HA-tagged SU(VAR)3-9 and V5-tagged RRP6 using the anti-HA antibody to pull down SU(VAR)3-9. The interaction with RRP6 was confirmed by western blotting.

In a previous study, we carried out co-immunoprecipitation experiments aimed at identifying interaction partners for the nuclear exosome of *D*. *melanogaster* in RNase A-digested nuclear extracts [34]. The proteins that co-immunoprecipitated with RRP6-V5 were identified by high-performance liquid chromatography/tandem mass spectrometry (LC/MS-MS). We detected a total of 418 proteins associated, directly or indirectly, with RRP6 when we set the false discovery rate to 0.01 (S1 Table). Previously known exosome interactors were detected in our study, including other components of the exosome, the transcription elongation factors SPT5 and SPT6 [20], and the insulator protein CP190 [35] (Fig 2A and S1 Table).

We carried out a gene-ontology (GO) analysis with the RRP6 interactors. Many of the GO terms associated with the RRP6-interacting proteins were related to known functions of the exosome in *D*. melanogaster. Interestingly, the term “chromatin organization” was also significantly enriched (Fig 2B).

Two heterochromatin proteins in the list of interaction partners drew our attention: SU(VAR)3-9 and RPD3 (Fig 2A and S1 Table). HP1a was also detected in two out of three LC/MS-MS experiments. These three proteins are functionally related to each other, and their interactions with RRP6 suggest a functional link between heterochromatin and RRP6.

We performed co-immunoprecipitation experiments followed by Western blotting to validate the interactions between RRP6 and heterochromatin factors found in the LC/MS-MS. In a first series of experiments, we used the S2-RRP6-V5 cells to validate the interaction of the V5-tagged RRP6 with RPD3 and HP1a (Fig 2C and 2D). The interaction between SU(VAR)3-9 and RRP6 was analyzed using the cell line that expressed RRP6-V5 and HA-tagged SU(VAR)3-9 (Fig 2E and S2A Fig). These experiments confirmed that RRP6 interacts, directly or indirectly, with RPD3, SU(VAR)3-9 and HP1a.

In agreement with the interactions reported above, the distribution of RRP6-V5 overlapped with that of SU(VAR)3-9 as shown by IF (S2B Fig), and a proximity ligation assay (PLA) confirmed the close association of SU(VAR)3-9 with RRP6 *in situ* (S2C Fig). The distributions of RRP6 and SU(VAR)3-9 in nuclear fractions were also very similar to each other (S2D Fig).

### RRP6 silences a subset of transposons and heterochromatic repeats

We depleted S2 cells of RRP6 by RNA interference (RNAi), and we carried out RNA-seq analysis to determine whether RRP6 plays a role in the expression of heterochromatic sequences. Total RNA preparations from cells treated with dsRNA complementary to either *Rrp6* (*RRP6 cells*) or GFP (control *GFP cells*) were ribosome-depleted and reverse transcribed using random primers, and the resulting cDNAs were sequenced to a depth of over 30 M reads per sample. The experiments included two independent biological replicates. The RRP6 levels were markedly reduced in the *RRP6 cells*, as expected (S3A Fig).

**Fig 3:**
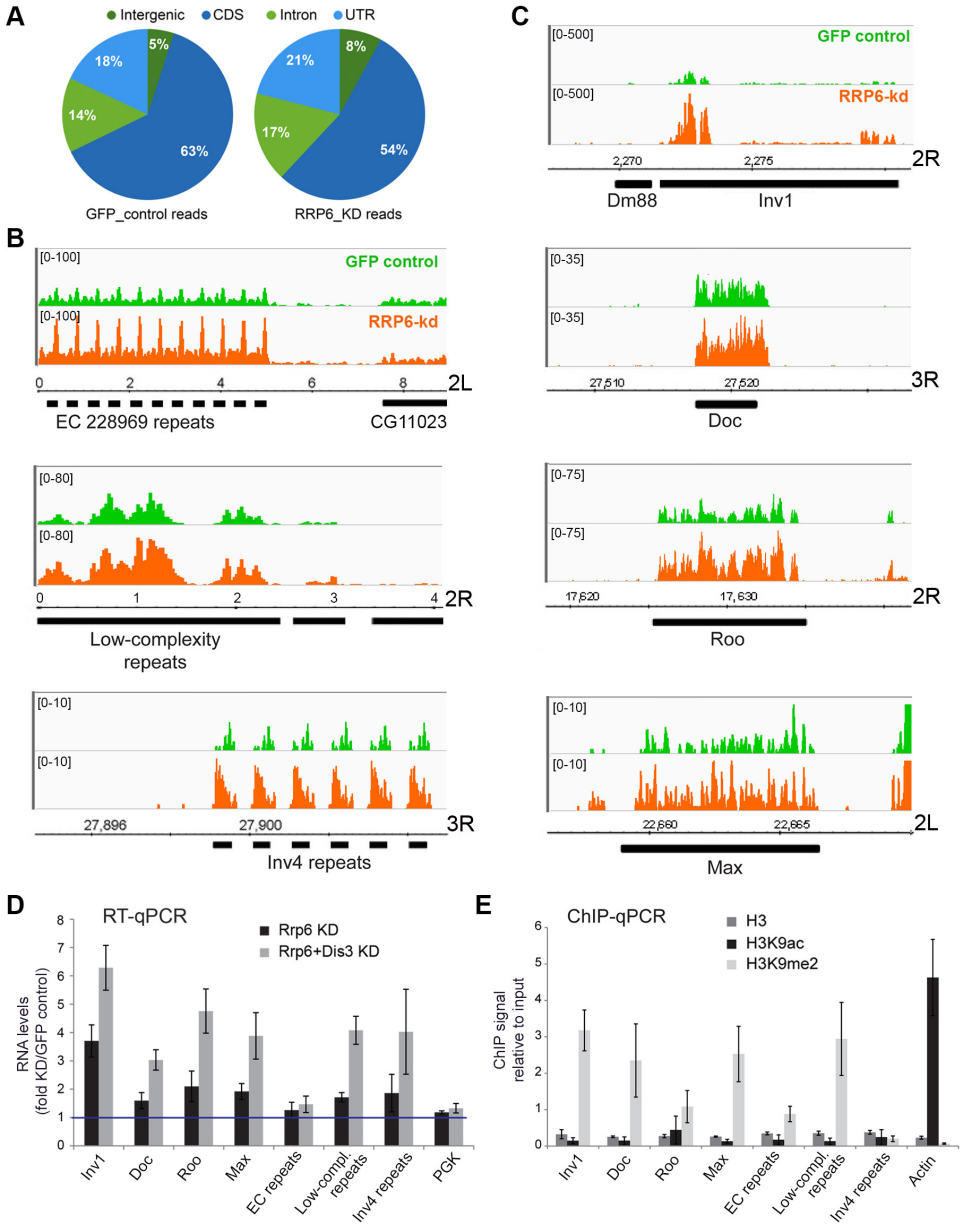
Genome-wide effects of RRP6 depletion on the transcriptome of S2 cells. The effects of RRP6 depletion on the steady-state expression levels were investigated by RNA-seq. Control experiments (GFP RNAi) were carried out in parallel and used as a reference. The expression levels in the control GFP cells and in RRP6-depleted cells expressed as reads per million (y-axis) are shown in green and orange, respectively. Genomic coordinates are indicated in the x-axis in B-C. (A) Pie diagram showing the effect of RRP6 depletion on the levels of different types of sequences, as indicated. (B) Examples of the effect of RRP6 depletion on the expression of repeat sequences. The upper and lower panels show subtelomeric regions of chromosome arms 2L and 3R, respectively, and the middle panel shows a region near the 2R centromere. (C) The effect of RRP6 depletion on the expression of selected transposon sequences. The genomic position of each sequence is indicated in the x-axis. (D) RNAi experiments were carried out to knock down RRP6 alone or RRP6 and DIS3 simultaneously. RNA was isolated and analysed by RT-qPCR using primer pairs designed to amplify selected sequences (the primer sequences are provided in the Supplementary Materials and Methods). The data was normalised to actin 5C RNA levels and expressed as a fold change compared to the levels observed in the GFP control. A protein-coding gene, *Pgk*, was analysed in parallel as a control. The histogram shows averages and standard deviations from three independent biological replicates. (E) ChIP experiments with antibodies against histone H3, H3K9ac, and H3K9me2 were carried out in untreated S2 cells to analyze the chromatin state of the selected genomic regions. Actin 5C was analyzed in parallel as a representative for euchromatin. The histogram shows averages and standard deviations from three independent biological replicates.

The analysis of RNA levels in the control *GFP cells* revealed the existence of significant expression over a large fraction of the genome, including heterochromatic regions that are rich in repetitive sequences, transposons and transposon fragments (Fig 3). The depletion of RRP6 did not destabilize the transcriptome on a global scale (S4A Fig), but affected the levels of expression of different types of transcripts in good agreement with the results from Kiss and Andrulis [36], Graham et al. [37] and Lim et al. [35]. The fraction of reads that mapped to intergenic sequences was significantly increased in *RRP6 cells* (Fig 3A, P < 0,0001), which is consistent with the role of RRP6 in the degradation of a large variety of non-coding and pervasive transcripts. Depletion of RRP6 caused an increase in the level of non-coding RNAs that are processed by the exosome, such as pre-rRNAs and some snoRNAs (S3B Fig). Depletion of RRP6 also increased the levels of transcripts derived from different types of heterochromatic repeats such as subtelomeric minisatellites and simple *gagaa* repeats (Fig 3B). Many transposons and transposon fragments showed increased transcript levels in *RRP6 cells* (Fig 3C, S4 Fig and S2 Table), including LTR retrotransposons, non-LTR retrotransposons and DNA transposons. However, not all transposons were affected (S4B Fig). Interestingly, we found that some elements of the same family showed increased or decreased transcript levels upon RRP6 depletion depending on their genomic insertion site (see for example *412* and *jockey* in S2 Table), which suggests that the genomic context has a stronger influence on the transcript levels than the transposon type. In summary, RRP6 is responsible for the silencing of a subset of transposons and repeats in the genome of *D*. *melanogaster*.

RT-qPCR experiments were carried out to validate the results from the RNA-seq experiments (Fig 3D). The ribonucleases RRP6 and DIS3 of *D*. *melanogaster* act on specific substrates, and have a certain redundancy [36]. We carried out, therefore, a double knockdown of RRP6 and DIS3. The increase in RNA abundances of selected retrotransposons and heterochromatic repeats was verified by RT-qPCR. Remarkably, an even greater stabilization of retrotransposon and repeat sequences was observed in cells depleted of both ribonucleases, whereas a protein-coding sequence (*Pgk* in Fig 3D) used as a control was not affected by the depletions. Depletion of DIS3 alone also resulted in increased levels of some heterochromatic transcripts (S3C Fig), which suggests that DIS3 contributes to the degradation of heterochromatic transcripts.

The heterochromatic transcripts derived from transposon and repeat elements that we analyzed in Fig 3D could be amplified by RT-qPCR using primer pairs that are 80–120 bp apart. This shows that these transcripts are relatively long non-coding RNAs or precursors to shorter RNA species.

The same sequences selected in Fig 3D were analyzed in control S2 cells by ChIP-qPCR using antibodies against histone H3, H3K9ac and H3K9me2 to investigate their chromatin state. The genomic regions that were upregulated in cells depleted of RRP6 and DIS3, with exception of the *Inv4 repeat* region in chromosome 3R, displayed low H3K9ac levels and high H3K9me2 levels (Fig 3E), which is characteristic of “classical/green” heterochromatin (according to the nomenclature of Filion et al. [38]).

### RRP6 degrades chromatin-associated transcripts and contributes to the compaction of heterochromatin

**Fig 4:**
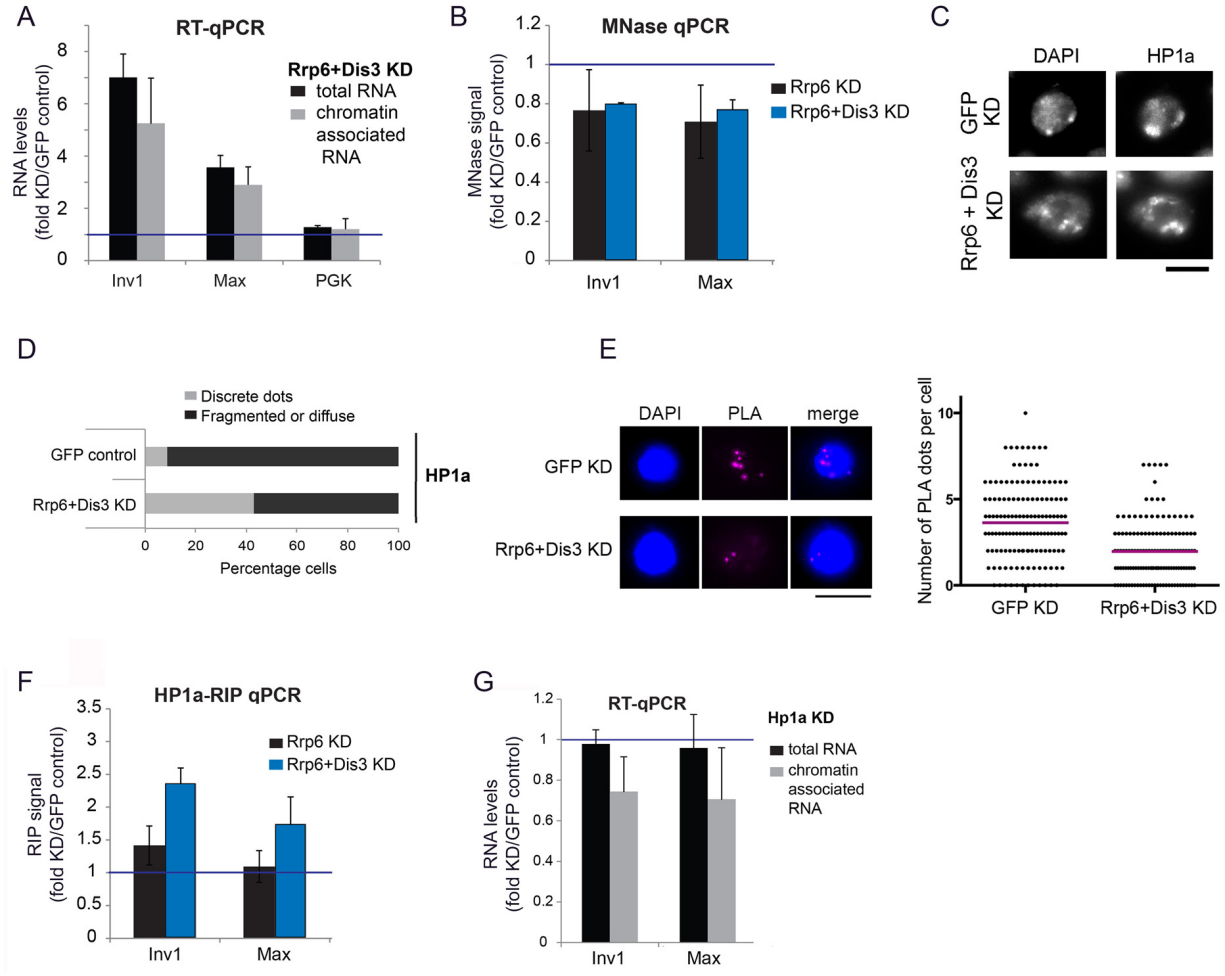
Depletion of exosome ribonucleases alters the compaction of the heterochromatin and increases the levels of heterochromatin-associated transcripts. (A) S2 cells were depleted of RRP6 and DIS3. Total RNA levels and chromatin-associated RNA levels were calculated for two selected heterochromatin sequences and a protein-coding gene (*Pgk*) as control. The data was normalized to actin 5C and expressed as a fold change relative to the GFP control. The histogram shows averages and standard deviations of three independent biological replicates. (B) MNase assays were carried out in S2 cells depleted of RRP6 or depleted of both RRP6 and DIS3. The digested chromatin was quantified by qPRC and compared to the corresponding chromatin in undigested samples. The values were normalized to a nucleosome-free region in the Hsp70 promoter (Petesch and Lis, 2008). The histogram shows the average values expressed as a fold change relative to the GFP control (blue line) calculated from four (for RRP6 KD) and two (for RRP6+DIS3 KD) independent biological replicates, respectively. The error bars represent standard deviations. (C) IF analysis of S2 cells after knock-down of RRP6 and DIS3. The figure shows examples of cells stained with antibodies against HP1a and counterstained with DAPI. The bar represents 5 μm. (D) Quantification of chromatin patterns in cells stained as in C. Cells were classified as either showing few discrete fluorescent dots, or fragmented or diffuse staining. The stacked bars show the percentage of cells in each class. (E) PLA analysis with antibodies against HP1a and histone H3 were carried out in S2 cells depleted of both RRP6 and DIS3, and in control cells treated in parallel with GFP-dsRNA. The images show examples of PLA staining (magenta) in cells counterstained with DAPI (blue). The graph shows the number of PLA dots per cell in each condition. The mean number of dots per cell (magenta line) was 3,63 in GFP control cells and 1,96 in cells depleted of Rrp6 and Dis3. The difference was highly significant (P<0.0001; two-tailed Mann Whitney test; n = 140 cells analyzed in each condition, from two independent experiments). (F) RIP experiments with an antibody against HP1a were performed to quantify the binding of HP1a with chromatin-bound RNAs derived from selected heterochromatic regions in control cells and in cells depleted of RRP6 and DIS3. The RIP signals of the knockdowns are expressed as fold changes relative to the GFP control samples (blue line). Averages and standard deviations from two biological replicates, each with two technical replicates, are presented. Note that the signals of the GFP samples were near background levels (S8 Fig). (G) S2 cells depleted of HP1a were analysed as in A. The data was normalised to actin 5C. Averages and standard deviations of three independent biological replicates are presented in the histogram.

Next we asked whether RRP6 and DIS3 act on chromatin-associated RNAs. We carried out RNAi experiments to knock down RRP6 and DIS3 in S2 cells, extracted chromatin as for ChIP experiments, and isolated the RNA from the chromatin preparations. We then carried out RT-qPCR reactions with primers specific for selected heterochromatic sequences (Fig 4A). The levels of chromatin-associated transcripts increased 3–6 fold in cells depleted of RRP6 and DIS3, and the increase was similar to that observed for total RNA isolated from the same samples (compare light and dark bars in Fig 4A). Control RT-qPCR reactions without reverse transcriptase (RT–) ruled out any significant DNA contamination (S5A Fig). In summary, RRP6 and DIS3 are responsible for the degradation of heterochromatic transcripts.

We then asked whether the compaction of the heterochromatin was affected by the depletion of RRP6 and DIS3, and we investigated this issue using a micrococcal nuclease (MNase) assay of crosslinked chromatin [39]. We knocked down RRP6 and DIS3 in S2 cells and carried out MNase assays (S5B Fig) with chromatin extracted from the RNAi-treated cells. In cells depleted of RRP6 and DIS3, the heterochromatin sequences were less represented than in control cells (Fig 4B), which shows that depletion of RRP6 and DIS3 renders the chromatin more accessible to MNase.

The experiments presented in Fig 4A and 4B suggest that depletion of RRP6 and DIS3 leads to a more accessible chromatin structure and increases the amount of chromatin-associated transcripts in heterochromatic regions of the genome. ChIP-qPCR experiments using an antibody against the large subunit of RNA polymerase II (Pol-II) showed a very slight increase in the density of Pol-II in the same heterochromatic regions (S6 Fig). This minor increase in Pol-II density can hardly account for the remarkable increase in the amount of RNA bound to chromatin (Fig 4A), which suggests that the accumulation of chromatin-bound RNA observed in the cells depleted of RRP6 and DIS3 is not due to increased transcription but to reduced ribonucleolysis.

We also analyzed the effects of depleting RRP6 and DIS3 on the overall organization of the chromatin in S2 cells by IF. In GFP control cells, DAPI staining revealed one or few prominent DAPI-intense regions that were stained by the anti-HP1a antibody (Fig 4C and 4D). HP1a was also located at these sites but was widely distributed throughout the nucleus. In cells depleted of RRP6 and DIS3, the DAPI-intense regions were fragmented or diffuse, and these fragmented DAPI-intense regions were more strongly stained by the anti-HP1a antibody than in the GFP control cells. We further probed changes in chromatin compaction using a PLA-based assay with antibodies against HP1a and histone H3. The rational of this assay was that treatments that result in a more open chromatin conformation would reduce the interaction between HP1 and histone H3 (S7 Fig). Depletion of RRP6 and DIS3 resulted in a significant reduction of H3-HP1a interaction (Fig 4E), in agreement with the results from the MNase assay. Altogether, the results reported above suggest that depletion of RRP6 and DIS3 affects the overall organization of the chromatin.

Given the ability of HP1a to bind RNA in both *Drosophila* and fission yeast [6], [7], and taking into account that depletion of RRP6 and DIS3 results in increased levels of chromatin-associated transcripts (Fig 4A), we hypothesized that depletion of RRP6 and DIS3 would also lead to increased levels of HP1a bound to chromatin-associated transcripts. We carried out RIP-qPCR experiments to analyze this possibility. In control *GFP cells*, the levels of chromatin-bound RNA were very low (close to background levels) at the selected heterochromatic sites (S8 Fig), and the depletion of RRP6 and DIS3 resulted in a pronounced increase of transcripts crosslinked to HP1a (Fig 4F).

Studies in *S*. *pombe* suggested that Swi6, the ortholog of HP1a, plays a role in the delivery of heterochromatic RNAs to the RNA degradation machinery [7]. We asked whether HP1a played a similar role in *D*. *melanogaster*, and analyzed the effect of depleting HP1a on the levels of chromatin-associated RNAs by RT-qPCR. If HP1a facilitated the degradation of heterochromatic RNAs, depletion of HP1a would render the heterochromatic RNAs more stable. The levels of RNAs associated with selected heterochromatic regions of the genome, however, decreased by approximately 30% (Fig 4G). This result is difficult to reconcile with a role for HP1a in RNA degradation. It is instead compatible with a previous study by Piacentini et al. [6], in which HP1a depletion caused a specific reduction of HP1a-target transcripts in *D*. *melanogaster*. In summary, our results suggest that HP1a does not contribute to the degradation of chromosomal transcripts in *D*. *melanogaster*, but instead stabilizes chromatin-associated RNAs probably by binding to them and thereby preventing their degradation.

**Fig 5:**
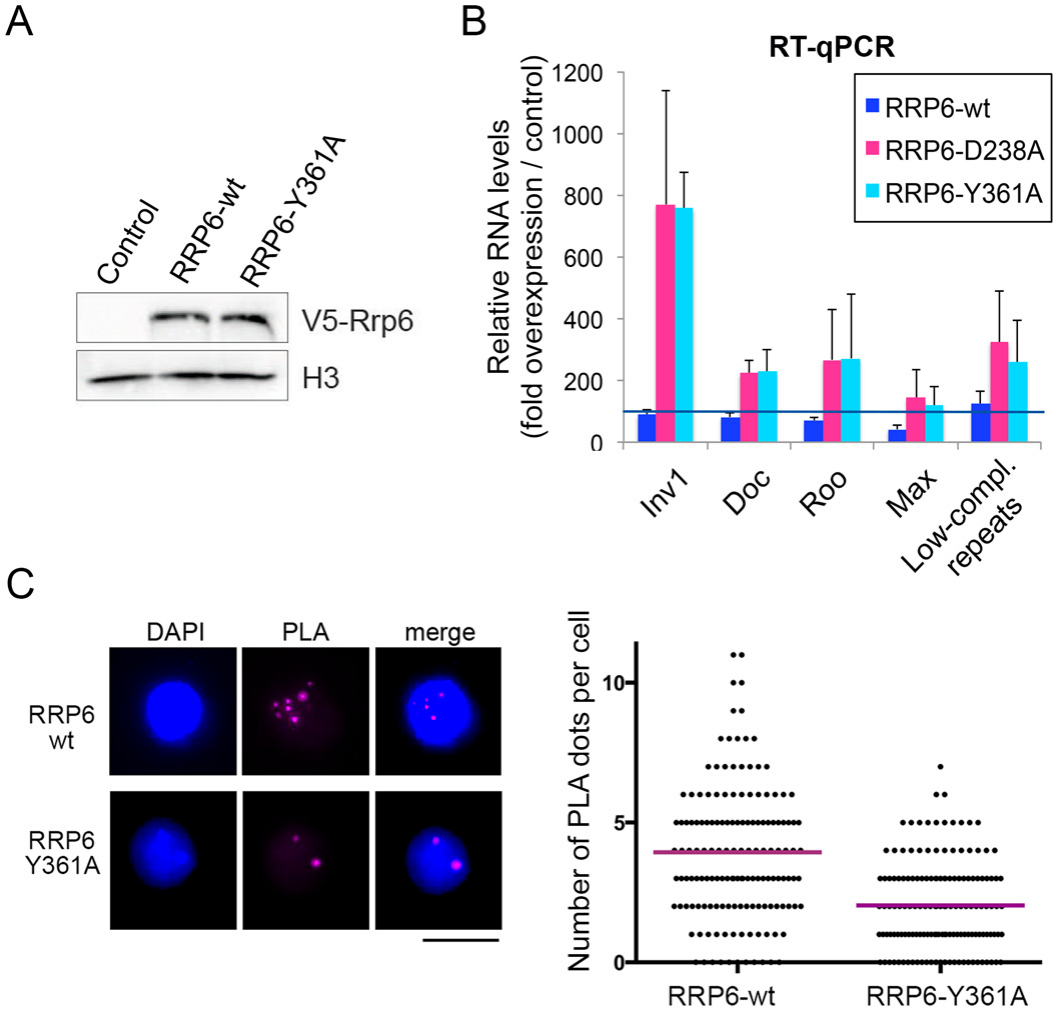
The catalytic activity of RRP6 is required for the silencing of transposon transcripts and for the maintenance of heterochromatin compaction. Wild-type RRP6-V5 or catalytically inactive mutants RRP6-Y361A-V5 and RRp6-D328A-V5 were expressed in S2 cells. Control cells that did not overexpress any protein were used in parallel for comparison. (A) Analysis of protein expression by Western blotting using an antibody against the V5 tag. Histone H3 served as loading control. (B) RT-qPCR analysis of transcript levels in cells that overexpress wither the wild-type RRP6-V5 or the catalytically inactive mutants. RNA was isolated and analyzed using primer pairs designed to amplify selected sequences, as indicated in the figure. The data was normalised to actin 5C mRNA levels and expressed as a fold change compared to the levels observed in the control cells (dark blue line). The histogram shows averages and standard deviations from three independent biological replicates. (C) PLA analysis of chromatin compaction using antibodies against HP1a and histone H3. The images show examples of PLA staining (magenta) in cells counterstained with DAPI (blue). The graph shows the number of PLA dots per cell in each condition. The mean number of dots per cell (magenta line) was 3,93 in the cells that overexpressed wild-type RRP6-V6 and 2,04 in cells that overexpressed RRP6-Y361A-V5. The difference was highly significant (P<0.0001; two-tailed Mann Whitney test; n = 150 cells analyzed in each condition, from two independent experiments).

In another series of experiments, we asked whether catalytically inactive RRP6 mutants could reproduce the effects observed upon depletion of RRP6 and DIS3. We used two different RRP6 mutants, RRP6-Y361A-V5 and RRP6-D238A-V5, that carry single amino acid substitutions in the active site and act as dominant negative mutants [28], [40]. These inactive RRP6 proteins were overexpressed in S2 cells, and the levels of transcripts derived from retrotransposon and repeat sequences were analyzed by RT-qPCR (Fig 5A and 5B). S2 cells overexpressing the wild-type RRP6-V5 protein were analyzed in parallel for comparison purposes. Overexpression of the dominant negative mutant proteins, RRP6-Y361A-V5 and RRP6-D238A-V5, led to a significant increase of transcript levels (Fig 5B), which supports the conclusion that RRP6 is involved in the degradation of these transcripts. We also analyzed whether the overexpression of a catalytically inactive RRP6 mutant had any detectable effect on chromatin compaction using the PLA-based assay described above. The PLA signal obtained in S2 cells that overexpressed the wild-type RRP6-V5 protein was significantly higher than that obtained in cells that overexpressed the RRP6-Y361A-V5 mutant (Fig 5C). Altogether, our present findings suggest that RRP6 is important for the degradation of heterochromatin-associated transcripts that, when stabilized, recruit HP1a and compromise the organization of the heterochromatin.

### SU(VAR)3-9 contributes to the association of RRP6 to a subset of genomic loci

**Fig 6:**
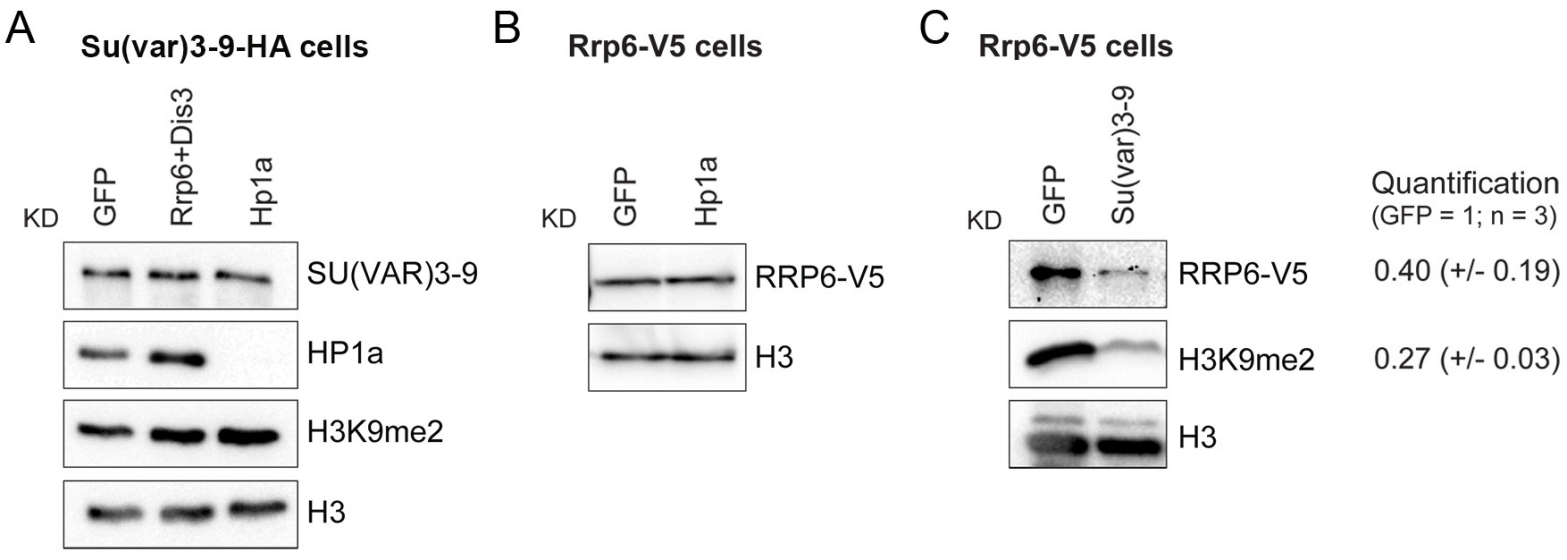
SU(VAR)3-9 depletion affects the association of RRP6 with chromatin. The association of selected proteins with the chromatin was analyzed by Western blotting using native chromatin preparations fractionated according to the scheme in Fig 1F. (A) Analysis of S2 cells that express the HA-tagged SU(VAR)3-9. The global levels of HP1a and SU(VAR)3-9 in the chromatin were analyzed in control cells (GFP) and in cells depleted of RRP6 and DIS3, or HP1a. The chromatin fractions were analysed using different antibodies, as indicated in the figure. An anti-HA antibody was used to detect SU(VAR)3-9. H3 and H3K9me2 served as controls. (B) Analysis of S2 cells that express the V5-tagged RRP6 depleted of HP1a. An anti-V5 antibody was used to detect RRP6 in the chromatin fractions. Depletion of HP1a does not affect the levels of RRP6 bound to the chromatin fraction. (C) Analysis of S2 cells that express the V5-tagged RRP6 depleted of SU(VAR)3-9. An anti-V5 antibody was used to detect RRP6. The quantification of the band intensities from three independent experiments is shown to the right. The standard deviations are given in parentheses. Histone H3 was used for normalization.

We carried out RNAi experiments to analyze the functional significance of the physical interactions between RRP6 and heterochromatin factors described in Fig 2. We depleted individual proteins in S2 cells (S9 Fig) and analyzed the effects of the depletion on the association of the remaining factors with the chromatin by semi-quantitative Western blotting. The chromatin preparations used for these experiments were native, non-fixed chromatin pellets prepared as in Fig 1E and 1F. These chromatin preparations were digested with RNase A to strip off RNA-bound proteins. A first series of RNAi experiments was carried out with S2 cells that expressed the HA-tagged SU(VAR)3-9. These experiments showed that the simultaneous depletion of RRP6 and DIS3 does not affect the association of SU(VAR)3-9 with the chromatin fraction nor the levels of H3K9me2 (Fig 6A and S10 Fig). In another series of RNAi experiments, we used S2 cells that expressed the V5-tagged RRP6 in order to analyze the association of RRP6 with chromatin. Depletion of HP1a did not cause a significant change in the association of RRP6 with the chromatin (Fig 6B). Instead, depletion of SU(VAR)3-9 reduced the amount of RRP6 in the chromatin fraction by 60% (Fig 6C) without a concomitant reduction in the levels of total RRP6 protein (S11 Fig). As expected, the SU(VAR)3-9 depletion also resulted in a very pronounced reduction of H3K9me2.

**Fig 7:**
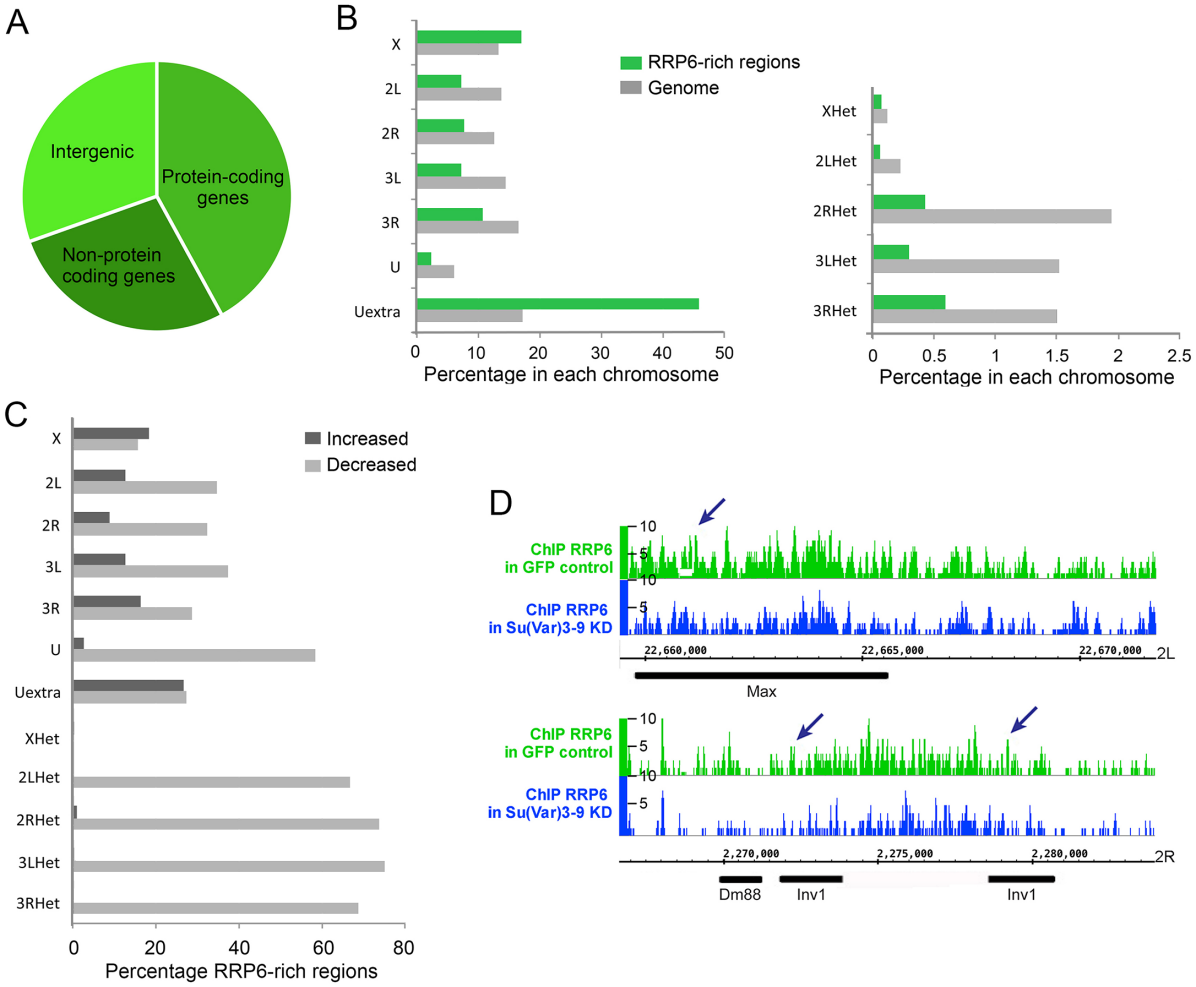
Depletion of SU(VAR)3-9 influences RRP6 genomic occupancy in S2 cells. ChIP-seq experiments were carried out using S2 cells that expressed V5-tagged RRP6 under low-induction conditions. Chromatin preparations from control GFP cells and from cells depleted of SU(VAR)3-9 were used for ChIP-seq using an anti-V5 antibody. (A) Pie diagram showing the association of RRP6-rich regions with different types of sequences in control cells. (B) Chromosome distribution of RRP6 expressed in control cells as percentage of RRP6-rich regions in each chromosome (green bars). Two different scales are shown due to the lower fraction of regions in the heterochromatic scaffolds compared to the rest of the chromosome arms. The grey bars indicate the fraction of the genome corresponding to each chromosome, for comparison. (C) Depletion of SU-VAR)3-9 affects RRP6 genomic occupancy. For each chromosome or scaffold, the number of RRP6-rich regions upregulated or downregulated is expressed as percentage of the number of changed regions relative to the number of regions in that same chromosome in control cells. The percentage of affected regions is much higher in heterochromatin. (D) RRP6 occupancy in the genomic regions analyzed in Fig 4. The arrows indicate the regions amplified in the qPCR assays.

The experiments presented in Fig 6 suggest that SU(VAR)3-9 is required for the association of RRP6 with chromatin. To confirm this observation and to identify the genomic regions in which the interaction of RRP6 with the chromatin is dependent on SU(VAR)3-9, we carried out ChIP-seq experiments to analyse RRP6 occupancy after SU(VAR)3-9 depletion (S12A Fig). We used the S2 cells that expressed V5-tagged RRP6 to pull down RRP6 with high specificity with an anti-V5 antibody, and we used low-induction conditions to avoid overexpression artefacts. As in previous experiments, cells treated in parallel with GFP-dsRNA were used as a control. Approximately 40% of the RRP6-rich regions were associated with protein-coding genes, and 30% with intergenic regions of the genome (Fig 7A). The average gene profile confirmed the association of RRP6 with gene promoters as previously reported [32], [35] (S12B Fig). In agreement with the IF staining shown in Fig 1A, RRP6 was broadly distributed in all the chromosomes (Fig 7B). The number of RRP6-rich regions was approximately twice as high in the X chromosome as in the autosomes (Fig 7B, green bars), which is interesting due to the dosage-compensation mechanisms that operate in the male X chromosome of S2 cells. At the molecular level, the enrichment of RRP6 in the X chromosome can perhaps be explained by the interaction between RRP6 and the MSL dosage-compensation complex [41]. The highest RRP6 occupancy was found in the so-called “Uextra” chromosome, which consists of unmapped heterochromatic scaffolds (see Uextra in Fig 7B). This could reflect a true association of RRP6 with heterochromatin, but the interpretation of this observation is problematic due to the high repeat content of these scaffolds.

The RRP6-rich regions identified by ChIP-seq overlapped with 967 gene loci. Approximately 20% of these RRP6-bound loci showed increased transcript levels in our RNA-seq analysis (RRP6/GFP log2 ratio > 1) (S3 Table). A likely interpretation is that these loci produce rapidly turned over transcripts. However, the majority of RRP6-rich regions do not show changed RNA levels in the RNA-seq experiment, which is compatible with the fact that most RRP6-bound genes are protein-coding genes that produce transcripts that are relatively stable and protected from RRP6-mediated degradation (see Discussion). This observation also implies that RRP6 recruitment does not necessarily imply transcript degradation, as previously proposed by Lim et al. [35].

Depletion of SU(VAR)3-9 reduced the total number of RRP6-rich regions by almost 30%. The RRP6-rich regions downregulated after SU(VAR)3-9 depletion were distributed in all the chromosomes, but were more represented in heterochromatic sequences (Fig 7C and S12C Fig). For example, 22 out of the 32 (69%) RRP6-rich regions detected in the 3RHet scaffold of control GFP cells were reduced after SU(VAR)3-9 depletion. Instead, the euchromatic chromosome X was the least affected by the depletion, with only 16% downregulated RRP6-rich regions.

Interestingly, the number of RRP6-rich regions located in annotated transposon loci were reduced by 56% in the SU(VAR)3-9-depleted cells, which reveals that the association of RRP6 with many transposons is dependent on SU(VAR)3-9. For example, the Max-element and Inv1 transposon sequences analysed in Figs 3–5 showed reduced RRP6 occupancy (Fig 7D). The SUVAR3-9 depletion also influenced RRP6 occupancy in many other regions, including not only transposons but also complex repeats and gene arrays such as histone and rRNA sequences (S12 Fig). Simple repeats mapped to the Uextra scaffold were instead not consistently affected by SU(VAR)3-9 depletion.

We classified the genes present in RRP6-rich regions in two groups: those located in genomic regions with reduced RRP6-occupancy upon SU(VAR)3-9 depletion (SUV-dependent regions) and those located in regions that were not significantly affected by the SU(VAR)3-9 depletion (SUV-independent regions). The SUV-dependent regions were characterized by relatively low levels of RRP6 occupancy compared to SUV-independent regions (S13 Fig). The transcripts produced in SUV-dependent regions showed significantly higher expression levels than the average of the transcriptome (higher average RNA-seq signals in control *GFP cells*, S4 Table). Moreover, the transcripts produced in SUV-dependent regions were more sensitive to RRP6 depletion, as shown by the fact that the fraction of transcripts from SUV-dependent regions that showed increased levels in RRP6-depleted cells (RRP6/GFP log2 ratio > 1) was significantly higher than the fraction of increased transcripts genome-wide (significant difference with P<0,0001, S4 Table). This observation is consistent with a model in which SU(VAR)3-9 contributes to the association of RRP6 to genomic loci that generate transcripts that are more actively turned over by RRP6 than the average of the transcriptome.

We also analyzed the effect of RRP6 depletion on the expression of RRP6-bound transposons, and found a positive correlation between RRP6 occupancy and RNA fold change upon RRP6 depletion (S14 Fig).

Recent studies revealed a role for piRNAs in the heterochromatin of somatic cells during early development [9], [15]. Sequences complementary to TAS1 and TAS2 piRNAs [42] showed reduced RRP6 occupancy in cells depleted of SU(VAR)3-9 (S15 Fig), which suggests that RRP6 is active in chromatin regions that can act as piRNA sources.

In summary, the results of the RRP6 ChIP-seq data indicate that SU(VAR)3-9 facilitates the association of RRP6 with chromatin, and in particular with repetitive heterochromatic sequences such as retrotransposons and retrotransposon fragments. RRP6 is also associated with non-heterochromatic sequences such as protein-coding genes and intergenic sequences, but the association of RRP6 with euchromatic regions of the genome is less dependent on SU(VAR)3-9 levels (see Discussion).

## Discussion

### An RRP6-dependent mechanism of heterochromatin maintenance in *D*. *melanogaster*

HP1a and SU(VAR)3-9 play a central role in the formation, spreading and maintenance of heterochromatin (reviewed in [43]). We reveal here a novel role for the SU(VAR)3-9 methyltransferase of *D*. *melanogaster* in the binding of RRP6 to the heterochromatin. Moreover, our results suggest that the local degradation of heterochromatin-associated transcripts by RRP6 is required to maintain the compaction of a subset of heterochromatic loci in the genome of *D*. *melanogaster*.

We have shown that RRP6 interacts physically with HP1a and SU(VAR)3-9, and that RRP6 is associated with a subset of heterochromatic regions of the genome. Less RRP6 is bound to the heterochromatin in cells with reduced levels of SU(VAR)3-9, which indicates that SU(VAR)3-9 contributes to the targeting of RRP6 to heterochromatin. Although the RNAi experiments do not reveal whether the effect of SU(VAR)3-9 knockdown on RRP6 occupancy is direct or indirect, the fact that RRP6 and SU(VAR)3-9 colocalize and can be co-immunoprecipitated suggests that SU(VAR)3-9 facilitates the recruitment of RRP6 to the heterochromatin, or stabilizes the interaction of RRP6 with other chromatin components, through a physical interaction.

We have focused our analysis on RRP6, and the existence of multiple exosome subcomplexes in cells of *D*. *melanogaster* [44] makes it difficult to establish whether the entire exosome has a role in the heterochromatin. However, two observations suggest that this is the case. Firstly, the simultaneous depletion of both catalytic subunits of the exosome, RRP6 and DIS3, gave additive effects on the levels of chromatin-associated RNAs and on the association of HP1a to heterochromatic RNAs. Secondly, we have previously shown that a fraction of RRP4, a core exosome subunit, is also associated with chromatin [34]. Altogether, these observations suggest that the entire exosome, not RRP6 alone, is targeted to heterochromatic loci through an interaction with SU(VAR)3-9.

Depletion of RRP6 or simultaneous depletion of RRP6 and DIS3 led to a local increase in heterochromatic transcripts associated with subtelomeric and pericentromeric regions, without a significant increase in the density of RNA Pol-II at those regions. This suggests that under normal conditions the RRP6 and DIS3 degrade pervasive RNAs that are transcribed from the heterochromatin.

Direct MNase assays and PLA-based assays designed to measure the compaction of the chromatin revealed that the depletion of the exosome ribonucleases loosens the structure of the heterochromatin in the regions that accumulate heterochromatic non-coding RNAs, without affecting the levels of H3K9 methylation or the association of SU(VAR)3-9 with the chromatin. In *S*. *pombe*, deletion of the *rrp6* gene leads to a derepression of heterochromatin, and this effect is partly due to the fact that in the absence of RRP6 activity, aberrant RNA species accumulate in *S*. *pombe* and recruit the siRNA machinery in competition with the RNAi-dependent pathways of H3K9 methylation [30], [45]. The situation is different in *D*. *melanogaster*, as no change in H3K9me2 or SU(VAR)3-9 recruitment occurred when RRP6 and DIS3 were depleted.

**Fig 8:**
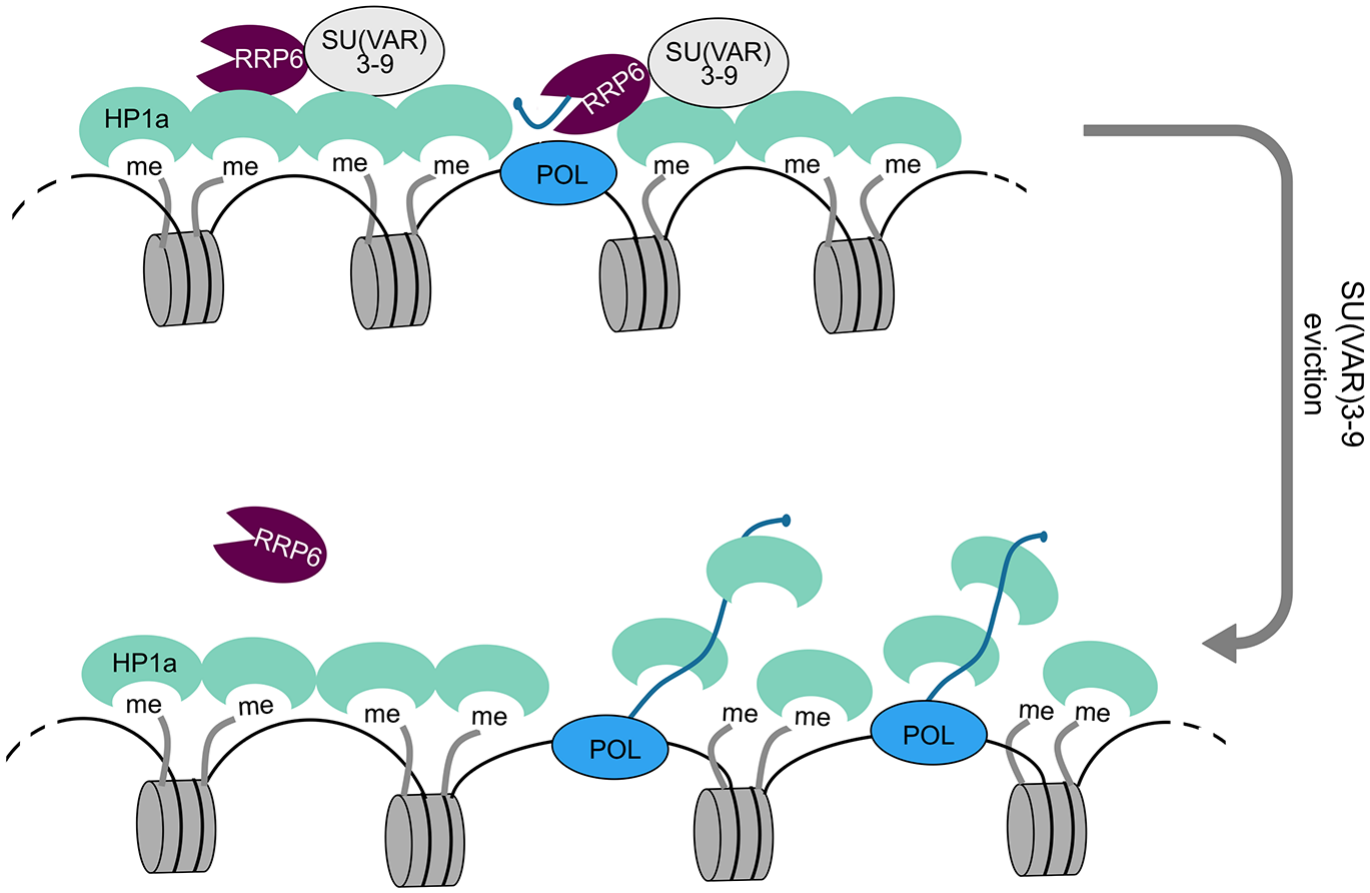
A model for the role of RRP6 in the maintenance of heterochromatin packaging. Heterochromatin domains are characterized by high levels of H3K9me2 and by the presence of HP1a and SU(VAR)3-9. Our results show that RRP6 interacts with SU(VAR)3-9 and that this interaction is important to tether RRP6 to the heterochromatin. Transcripts derived from sporadic transcription of heterochromatic repeat sequences are kept at low levels by RRP6 degradation. Failure to degrade such transcripts results in increased levels of chromatin-associated transcripts, increased binding of HP1 to the chromatin-associated transcripts, and chromatin decondensation.

What is then the mechanism by which the exosome ribonucleases influence the compaction of the heterochromatin in *D*. *melanogaster*? The HP1a ortholog in *S*. *pombe*, Swi6, is an RNA-binding protein, and non-coding RNAs can cause the eviction of Swi6 from the *S*. *pombe* heterochromatin by competing with H3K9me for Swi6 [7], [46]. The HP1a protein of *D*. *melanogaster* interacts with several RNA-binding proteins and can bind directly to RNA [6]. We have shown that depletion of RRP6 and DIS3 results in increased levels of non-coding transcripts associated with heterochromatin in *D*. *melanogaster* cells. HP1a-RIP signals at selected heterochromatic loci are also increased in cells depleted of RRP6 and DIS3. Altogether, these observations are consistent with a model in which RRP6, and perhaps also DIS3, participate in the degradation of heterochromatic non-coding RNAs that, if stabilized, would outcompete the binding of HP1a to the methylated H3K9 and would thereby disrupt the packaging of the heterochromatin (Fig 8).

### Specialized protein-protein interactions target RRP6 to different chromatin environments

RRP6 and the exosome act on many different types of transcripts and participate in many essential biological processes [17], [21], [22]. The existence of multiple mechanisms to target RRP6 to different types of transcripts, or even to different nuclear compartments, is thus not unexpected. The association of the exosome-or exosome subunits- with genes transcribed by RNA polymerase II (Pol-II) is mediated by interactions with different types of proteins. Co-immunoprecipitation experiments in *D*. *melanogaster* identified SPT5 and SPT6, two transcription elongation factors, as interaction partners for the exosome, which led to the proposal that the exosome is tethered to the transcription machinery during transcription elongation [20]. In *D*. *melanogaster*, the exosome is also tethered to protein-coding loci through interactions with the hnRNP protein HRP59/RUMP [34]. In human cells, a NEXT complex containing MTR4, the Zn-knuckle protein ZCCHC8, and the putative RNA binding protein RBM7 mediates an interaction between the exosome and Pol-II transcripts through the nuclear cap-binding complex [47], [48]. In many cases, these intermolecular interactions target the exosome to genomic loci that produce relatively stable transcripts, for instance protein-coding transcripts or stable non-coding RNAs. In these loci, the role of the exosome is primarily linked to RNA surveillance, not turnover.

Much less is known about the mechanisms that target the exosome or its individual subunits to non-protein coding RNAs in the heterochromatin. Our study of the RRP6 interactome in cells of *D*. *melanogaster* has revealed interactions between RRP6 and heterochromatin factors, and has established an important role for SU(VAR)3-9 in determining RRP6 occupancy. Depletion of SU(VAR)3-9 has a profound effect on the association of RRP6 with a subset of chromatin regions, including many transposon loci. Our present findings suggest that these regions, that we refer to as “SUV-dependent”, produce transcripts that are actively degraded by RRP6. SU(VAR)3-9 has less impact on the targeting of RRP6 to euchromatic protein-coding genes, where interactions with the Pol-II machinery and with mRNA-binding proteins play instead a decisive role. Altogether, the picture that emerges from many studies is that specialized protein-protein interactions target RRP6 to specific genomic environments where RRP6 participates in the processing, surveillance or degradation of specific RNA substrates.

## Materials and Methods

### Culturing conditions

S2 cells were cultured at 28°C according to the instructions of the *Drosophila Expression System* manual from Invitrogen. Stably transfected cells were cultivated in Schneider’s medium containing 300 μg/ml hygromycin B (Invitrogen) or 2 μg/ml puromycin (Invitrogen). The S2 cells stably transfected with plasmids for expression of V5-tagged RRP6 (S2-RRP6-V5) and mutant RRP6 (S2-RRP6-Y361A-V5) are described in [34] and [28], respectively.

### Plasmid preparation and stable transfection of S2 cells

The open reading frame of SU(VAR)3-9 was amplified by PCR and cloned into the pMT-puro Drosophila expression vector (Addgene). Detailed cloning information is given in the Supplementary Materials and Methods (S1 Text). The plasmid was transfected into S2 cells that already carried an expression construct for RRP6-V5. The Calcium Phosphate Transfection kit (Invitrogen) was used for the transfection.

A single amino-acid substitution D238A in *RRP6-V5* was made by oligonucleotide-directed site-specific mutagenesis using the Site-directed mutagenesis kit (Invitrogen) on the pMT-Rrp6 plasmid [34] as described in [28]. The sequences of the oligonucleotides used were 5'-CAGATCGCCATAGCTGTGGAGCACCACTC-3' (oligo Ae123) and 5'-GAGTGGTGCTCCACAGCTATGGCGATCTG-3' (oligo Ae124).

### Antibodies

The following antibodies were used in this study: anti-V5 (R960-25, Invitrogen), anti-HP1a (C1A9, Developmental Hybridoma Bank), anti-HA (ab9110, abcam), anti-RPD3 (ab1767, abcam), anti-tubulin (ab44928, abcam), anti-H3 (ab1791, abcam), anti-H3K9ac (ab10812, abcam), anti-H3K9me2 (ab1220, abcam and 302–32369, Wako), anti-CTD (ab5408, abcam). The anti-RRP6 antibody was a generous gift from E. Andrulis. Secondary antibodies conjugated to FITC, Texas Red or colloidal gold were purchased from Dako and Jackson ImmunoResearch Laboratories.

### Immunoprecipitation and high-performance liquid chromatography/tandem mass spectrometry

Immunoprecipitation and LC/MS-MS were performed as described in [34] using nuclear protein extracts prepared from S2 cells that expressed V5-tagged RRP6, or from “empty” control cells that were cultivated under the same conditions but did not express any V5-tagged protein. An enrichment probability value compared to negative controls was calculated for each protein identified, using a standard error model. More information about the mass spectrometry and bioinformatics analysis is given in [34] and in the Supplementary Materials and Methods (S1 Text). Significant interactions were identified using a false discovery rate (FDR) threshold of 0.01, as well as a minimum fold-difference of 2 compared to “empty” cells. Functional annotation and GO-enrichment analysis were performed using DAVID [49], with the parameter Ease set to 0.01.

### Co-immunoprecipitation experiments

S2 cells were resuspended in lysis buffer and homogenized using a glass homogenizer with a tight pestle B. After centrifugation, the supernatant (corresponding to the cytoplasm) was discarded. The pellet was resuspended in PBS that contained complete protease inhibitor, sonicated four times for 3–4 pulses each, and centrifuged at 16,000g for 15 min at 4°C. The supernatant was used for co-immunoprecipitation according to standard procedures. For details, see the Supplementary Materials and Methods (S1 Text). The samples were analysed by SDS-PAGE and western blotting.

### Fractionation of the cell nucleus

Nuclei were isolated from S2 cells and fractionated as described in [33]. The detailed protocol is provided in the Supplementary Materials and Methods (S1 Text). In short, S2 cells were resuspended in lysis buffer containing 0.2% Nonident P40 substitute, homogenized and centrifuged. The pellet (nuclei) was dissolved in PBS, sonicated and centrifuged again. The proteins of the supernatant were the *soluble* nuclear fraction. The pellet was digested with RNase A, and centrifuged. The proteins of the supernatant and pellet were the *chromosomal RNP* and *chromatin* fractions, respectively.

### Immunofluorescent staining of S2 cells and polytene chromosomes

S2 cells and polytene chromosome squashes were prepared and immunostained following standard methods as described in the Supplementary Materials and Methods (S1 Text). The slides were examined in either a Axioplan fluorescence microscope (Carl Zeiss) or an LSM 510 laser confocal microscope (Carl Zeiss). Co-localization was analyzed using the *Profile* function of the LSM 510 software by drawing a test line along the region of interest and measuring the relative fluorescence intensity along the line (in arbitrary units). Proximity ligation assays (PLA) were carried out using Duolink probes (Olink Bioscience) according to the procedures recommended by the manufacturer.

### Proximity ligation assay (PLA)

Cells were fixed with 3.7% formaldehyde in PBS for 10 min and permeabilized with 0.1% Triton X-100 for 15 min at room temperature. A blocking solution of 3% BSA in PBS was added for 40 min followed by 1 h incubation of the primary antibodies diluted in 0,3% BSA. The proximity ligation assay (PLA) was carried out using the Duolink PLA *in situ* kit (Olink) following the manufacturer’s protocol. The preparations were analyzed in an Axioplan fluorescence microscope (Carl Zeiss).

### Immuno-electron microscopy

S2 cells were fixed in 4% paraformaldehyde, cryoprotected, frozen by immersion in liquid nitrogen and cryosectioned. The primary antibodies were anti-RRP6 antibody [20] and anti-HP1a antibody. The secondary antibodies were conjugated to 6 nm and 12 nm gold particles (Jackson ImmunoResearch Laboratories). After immunolabelling, the sections were stained with 2% aqueous uranyl acetate, embedded in polyvinyl alcohol and examined in a FEI Tecnai G2 electron microscope at 80 kV.

### RNA interference in S2 cells

RNA interference was carried out essentially as described in [50]. More information and primer sequences are given in the Supplementary Materials and Methods (S1 Text).

### Real-time PCR (qPCR)

Real-time PCR (qPCR) was performed in a Qiagen RotorGene Q with KAPA SYBR Fast qPCR Master Mix (KAPA Biosystems). All primers used for qPCR are described in the Supplementary Materials and Methods (S1 Text). The qPCR assays followed the MIQE guidelines for primer design [51] and all primer pairs fulfilled quality criteria according to amplification and melting curves. Normalization of the qPCR data is described in the corresponding figure legend.

### RNA-seq

S2 cells were treated with dsRNA to knock down RRP6. Control cells were treated in parallel with dsRNA that was complementary to GFP. Total RNA was ribosome depleted and used to construct random-primed cDNA libraries for next-generation sequencing. The libraries were prepared and sequenced by GATC-Biotech AB on a HiSeq2500 sequencer to a depth of at least 30 M single-end reads per sample. RNA samples from two independent experiments were sequenced in parallel, each with a GFP control sample. Illumina fastq files were inspected with FastQC to assess quality of the reads. High quality reads were mapped with TopHat2 [52] to the *Drosophila melanogaster* genome assembly, build BDGP6 (Dm3). Details on sample preparation and data analysis are provided in the Supplementary Materials and Methods (S1 Text). The RNA-seq results were visually inspected against the April 2006 version of the *Drosophila melanogster* genome with the Integrative Genomics Viewer (https://www.broadinstitute.org/software/igv/) [53]. The data generated in this study are available at NCBI’s Gene Expression Omnibus (accession number GSE66640).

### Micrococcal nuclease (MNase) experiment

The MNase experiments were performed essentially as described by Petesch and Lis [39]. A detailed description is provided in the Supplementary Materials and Methods (S1 Text).

### ChIP-qPCR and ChIP-seq

ChIP-qPCR experiments were performed as described in [54]. A synthetic DNA-antibody complex was used as external reference to normalise the ChIP data [54]. RRP6-V5 cells were treated with dsRNA for either GFP (controls) or Su(Var)3-9, and used for ChIP-seq with anti-V5 antibody. DNA libraries were prepared by Zymo Research Epigenetics Services and were sequenced on a HiSeq2500 sequencer. ChIP-seq data are available at NCBI’s Gene Expression Omnibus (accession number GSE66640). Details on data analysis are provided in the Supplementary Materials and Methods (S1 Text).

### RNA immunoprecipitation (RIP) experiment

Cells were fixed and the chromatin was extracted as for ChIP experiments. After sonication of the chromatin, the genomic DNA was degraded for 30 min at 37°C with 100 U DNases (Thermo Scientific) and the sample was used for immunoprecipitation as described in the Supplementary Materials and Methods (S1 Text). RNA was extracted from the immunoprecipitated material with Trizol (Invitrogen) and the total RNA was transcribed into cDNA and analyzed with qPCR.

## Supporting Information

S1 Fig Partial colocalization of endogenous HP1a and RRP6 in *Drosophila* S2 cells. Immunofluorescent staining of fixed S2 cells with antibodies against RRP6 (green) and HP1a (red). The fluorescence profile in the right part of the image shows the co-variation of the fluorescent signals in each channel along an axis through the nucleus (white arrow). The distributions of RRP6 and HP1a are different, but both proteins colocalize in some regions of the nucleus. The blue arrows show regions of co-localization.
(PDF)

S2 Fig Partial colocalization of SU(VAR)3-9 and RRP6 in *Drosophila* S2 cells. (A) Simultaneous expression of V5-tagged RRP6 and HA-tagged SU(VAR)3-9 in S2 cells. S2 cells stably transfected with plasmids for expression of RRP6-V5 and the SU(VAR)3-9-HA (see Materials and Methods for details) were induced with different concentrations of CuSO_4_, and the expressions of the RRP6-V5 and the SU(VAR)3-9-HA proteins were detected by Western blotting using the anti-V5 antibody and the anti-HA antibody, respectively. Tubulin served as a loading control. (B) Colocalization of SU(VAR)3-9 and RRP6 in *Drosophila* S2 cells. Immunofluorescent staining of S2 cells that expressed HA-tagged SU(VAR)3-9 and V5-tagged RRP6. The cells were fixed and stained with antibodies against HA (green) and V5 (red). The fluorescence profile in the right part of the image shows the co-variation of the fluorescent signals in each channel along an axis through the nucleus (white arrow). The distributions of RRP6 and SU(VAR)3-9 are different, but both proteins colocalize in some regions of the nucleus (blue arrow). (C) Proximity ligation assay (PLA) showing close proximity between SU(VAR)3-9 and RRP6 in *Drosophila* S2 cells. S2 cells that expressed HA-tagged SU(VAR)3-9 and V5-tagged RRP6 were double stained with antibodies against HA and V5, and the proximity was assayed using DuoLink probes (red signal). The cells were counterstained with DAPI (blue). (D) Nuclear fraction analysis of the Rrp6—Su(var)3-9 cells. Protein expression in the Rrp6—Su(var)3-9 cells was induced with 200 μM CuSO_4_ overnight. The cells were harvested and the nuclei were isolated as described in Materials and Methods. The nuclei were separated into soluble (nucleoplasm), chromosomal RNP, and chromatin fractions according to the scheme shown in Fig 2A. The different fractions were analyzed by SDS-PAGE and Western blotting.
(PDF)

S3 Fig Depletion of exosome ribonucleases affects the levels of different types of transcripts in S2 cells. (A) Analysis of RRP6 knockdown efficiency. S2 cells were treated with long dsRNA against Rrp6, or against GFP as a control, to deplete the cells of RRP6 protein (see Supplementary Materials and Methods, S1 Text, for details). The cells were harvested 96 hours after the first dsRNA administration. The efficiencies of the knockdown treatments were determined by SDS-PAGE and Western blotting using an antibody against RRP6. Tubulin served as a loading control. The red asterisk in the figure indicates a background signal of the antibody. (B) RRP6 depletion resulted in pre-rRNA processing defects. RRP6 depletion inhibits the trimming of the 3' end of the pre-rRNA CR41608, as shown by the increased amount of RNA complementary to the 3' end of the gene (left panel). RRP6 is also needed for the processing of other functional RNAs, including snoRNAs, and depletion of RRP6 leads to increased levels of snoRNA transcripts (right panel). (C) Depletion of DIS3 resulted in increased levels of some heterochromatic transcripts. In S2 cells depleted of DIS3 (and GFP as control), the RNA levels of two selected heterochromatic transposon sequences (Inv1 and Max) were measured by RT-qPCR. The data from DIS3-KD cells and GFP control cells were normalized to Actin 5C and the results are expressed as a fold change comparing the levels obtained in the DIS3-KD with the levels in the GFP control (the blue line indicates no change). The bars represent averages and the error bars standard deviations from three independent biological replicates.
(PDF)

S4 Fig The effect of RRP6 depletion on the S2 transcriptome. S2 cells were treated with long dsRNA against Rrp6 to deplete the cells of RRP6 protein, or against GFP as a control, as in S3 Fig. Expression levels were analysed by RNA-seq and the figure shows comparisons between knockdown of RRP6 and the GFP control samples. (A) All ORF and ncRNAs (n = 13272). 1534 genes showed increased expression levels (average log2 ratio > 1, blue). 213 genes showed decreased expression levels (average log2 ratio < 1, orange). (B) Transposable elements (n = 1572). 75 transposons showed increased expression levels (average log2 ratio > 1, blue). 9 transposons showed decreased expression levels (average log2 ratio < 1, orange). r indicates Pearson’s correlation coefficient.
(PDF)

S5 Fig Control experiments: RT(-) controls and micrococcal nuclease (MNase-qPCR) assays. (A) Analysis of genomic DNA contamination in cDNA samples. RNA samples (total RNA and chromatin-associated RNA) were treated with DNAse and reverse transcribed into cDNA as described in the Materials and Methods. RT (-) control reactions were processed in parallel without adding reverse transcriptase to the RT reaction mixture. The Actin 5C levels in RT(+) and RT(-) samples were analyzed by RT-qPCR and compared to each other to quantify possible genomic DNA contamination in the cDNA samples. The table shows data from three independent experiments (EXP I, II, III). The figures are the percentages of genomic contamination in the cDNA samples. (B) MNase experiments showed nucleosome density on analyzed regions of the genome. Chromatin prepared from S2 cells was treated with 20 U MNase or with no MNase (for details see the Materials and Methods). After DNA purification, the MNase-treated samples were compared to untreated samples by qPCR. A low value in a specific genomic region means that this region is nucleosome-free (or less condensed), while a high value in a genomic region points towards a more compact chromatin structure. Hsp70 was used as a control since the promoter is nucleosome-free and a positioned nucleosome is present in the coding region [39]. The figure shows averages and standard deviations from three independent experiments. These control experiments based on the analysis of the Hsp70 promoter showed that open chromatin regions were more accessible to MNase than compact regions, and are therefore less represented in the digested chromatin preparations. These experiments also showed that the three analyzed heterochromatic regions were approximately four times more represented than the open Hsp70 used as a control, which is consistent with the heterochromatic nature of these regions.
(PDF)

S6 Fig ChIP-qPCR analysis of Pol-II in cells depleted of RRP6 and DIS3. ChIP experiments with an antibody against RNA polymerase II in S2 cells depleted of RRP6 and DIS3. ChIP signals were calculated relative to the corresponding input sample. An external standard was used for normalization as described by Eberle et al. (2012) [54]. The histogram shows the average signals and standard deviations of the fold change obtained when comparing the ChIP signals in the Rrp6+Dis3 KD with those in the control GFP KD. Data from four independent biological replicates.
(PDF)

S7 Fig A PLA-based assay to analyse changes in chromatin compaction. S2 cells were analysed by PLA with antibodies against HP1a and histone H3. The rationale of the assay is that a more open chromatin conformation reduces the interaction between HP1a and histone H3. (A) PLA analysis of control S2 cells. The slides were counterstained with DAPI (blue). The figure shows negative control reaction with each of the antibodies separately. PLA signals (*magenta dots*) were observed only in the presence of both antibodies and were restricted to the cell nucleus, as expected. (B) PLA analysis of cells depleted of SU(VAR)3-9. A control experiment was carried out to assess the suitability of the H3-HP1a PLA assay to detect changes in chromatin compaction. The number of PLA dots per cells in cells depleted of SU(VAR)3-9 was compared to that observed in GFP control cells. The figure shows representative examples of the results obtained in each condition. (C) Quantitative analysis of the results of the experiment described in B. The graph shows the results obtained from 265 cells analysed in each condition. Depletion of SU(VAR)3-9 reduced more than three-fold the number of PLA dots per cell. This difference was highly significant (P<0.0001 in a two-tailed, nonparametric Mann Whitney test). The magenta bars in the graph indicated the mean value in each condition. Mean and SEM values for each condition are provided below the graph.
(PDF)

S8 Fig HP1a-RIP experiments showing the background levels. RIP experiments were performed with an anti-HP1a antibody (black bars) and no-antibody controls (neg; grey bars) (for details see Materials and Methods). The immunoprecipitated RNA was reverse transcribed into cDNA and specific sequences (Inv1, Max, and Actin) were analyzed by RT-qPCR. The data was set relative to the GFP sample. The figure shows averages and standard deviations obtained from two independent biological replicates, each quantified in duplicate. HP1a bound to RNA in the chromatin is undetectable in the GFP controls (RIP signals for HP1a are background levels), but a significant increase was measured in cells depleted of exosome ribonucleases (RIP signals above background levels).
(PDF)

S9 Fig Analysis of knockdown efficiencies. (A) S2 cells were treated with dsRNA against GFP (control), Hp1a, or Su(var)3-9. To analyze the knockdown efficiencies, total RNAs were purified and were reverse transcribed into cDNA, and the resulting cDNAs were analyzed by qPCR. The RNA levels were normalized to Actin 5C and expressed as a fold change compared to the GFP control. Averages and standard deviations of three (for Hp1a) and two (for Su(var)3-9) independent experiments are shown in the figure. (B) The Hp1a knockdown was also determined by Western blotting. Tubulin and H3K9me2 served as loading controls.
(PDF)

S10 Fig H3K9me2 analysis in cells depleted of RRP6 and DIS3. Depletion of RRP6 and DIS3 did not cause any significant change in the levels of H3K9me2 at the analysed sites. Chromatin was harvested from S2 cells depleted of GFP (as a control), RRP6, or RRP6 and DIS3 together. ChIP experiments were performed with the anti-H3K9me2 antibody. The ChIP signals were calculated relative to the corresponding input and normalized to an external reference DNA sequence that was added in the beginning of the experiment (see Eberle et al., 2012) [54]. The histogram shows the average signals and standard deviations expressed as a fold change compared to the GFP control from four independent biological replicates for RRP6 KD (black bars) and three for the double knockdown (grey bars), each with one qPCR run.
(PDF)

S11 Fig Analysis of RRP6 in S2 cells depleted of SU(VAR)3-9. (A) S2-RRP6-V5 cells were treated with dsRNA against GFP (control) or Su(var)3-9. Nuclei were isolated and fractionated, and the soluble and chromatin fractions were analysed by Western blotting. Reduced RRP6 signal was observed in the chromatin pellet but not in the soluble fraction, which suggests that the overall levels of expression of RRP6 are not reduced upon Su(Var)3-9 KD. (B) S2 cells were treated with dsRNA against GFP (control) or Su(var)3-9. Whole-cell extracts were prepared and analyzed by Western blotting.
(PDF)

S12 Fig RRP6 ChIP-seq analysis. ChIP-seq experiments were carried out using S2 cells that expressed V5-tagged RRP6 under low-induction conditions. The expression of Su(Var)3-9 was knocked down using dsRNA. Control cells were treated in parallel with GFP-dsRNA. (A) ChIP-seq parameters. The table indicates the number of reads mapped to the genome, the number of RRP6-rich regions determined by MACS version 2.1.0 with a q-value cutoff of 5.00e-02, the average length and fold enrichment of the peaks in each condition. (B) The meta-gene distribution of RRP6 computed by MACS2, including 1 kb upstream of the transcription-start site (TSS) and 1 kb downstream of the transcription-termination site (TTS). (C) Upregulated and downregulated RRP6-rich regions in the different chromosomes of *D*. *melanogaster*. The table also shows the chromosome distribution of RRP6-rich regions in control, GFP-treated cells. Upregulated RRP6-rich regions are regions that are found in cells depleted of Su(Var)3-9 but not in GFP control cells. Downregulated regions are those that are present in GFP control cells but not in Su(Var)3-9-depleted cells. Peaks with at least 50% length overlap were considered to be the same peak and were not considered changed in this analysis. (D) Examples of RRP6 occupancy in selected loci corresponding to different types of sequences, as indicated in the figure. The data was visualized and the images generated using the Integrated Genome Browser v. 8.1.11.
(PDF)

S13 Fig The effect of RRP6 depletion on the levels of transcripts originated from RRP6-bound genes. Scatter plot showing the levels of RRP6-occupancy in S2 cells (measured by ChIP-seq in control GFP cells) and the effect of RRP6 depletion (measured by RNA-seq in cells treated with RRP6-dsRNA compared to control GFP cells) for each transcript. Transcripts from SUV-dependent and SUV-independent genes are represented in different colors, as indicated. The Spearman’s rank correlation coefficient is indicated (r).
(PDF)

S14 Fig The effect of RRP6 depletion on the levels of transcripts originated from RRP6-bound transposons. Scatter plot showing the levels of RRP6-occupancy in S2 cells (measured by ChIP-seq in control GFP cells) and the effect of RRP6 depletion (measured by RNA-seq in cells treated with RRP6-dsRNA compared to control GFP cells) for each transposon. The Spearman’s rank correlation coefficient is indicated (r).
(PDF)

S15 Fig RRP6 occupancy in two genomic regions that generate piRNA during early development. ChIP-seq experiments were carried out using S2 cells that expressed V5-tagged RRP6 under low-induction conditions, as in S8 Fig. The expression of Su(Var)3-9 was knocked down using dsRNA. Control cells were treated in parallel with GFP-dsRNA. The image shows examples of RRP6 occupancy in two selected genomic regions that are known to generate piRNAs during early development (Yin & Lin, 2007; Nature 450:304–308). The data was visualized and the images generated using the Integrated Genome Browser v. 8.1.11.
(PDF)

S1 Table List of RRP6 interactors. (PDF)

S2 Table List of most increased and most decreased transposon insertion sites. (PDF)

S3 Table RRP6-bound genes showing differential expression upon RRP6 KD. (PDF)

S4 Table Effect of RRP6 depletion on the expression of RRP6-bound genes. (PDF)

S1 Text Supplementary Materials and Methods. (DOCX)
