# Supplementary figures and images for: An Interaction between RRP6 and SU(VAR)3-9 Targets RRP6 to Heterochromatin and Contributes to Heterochromatin Maintenance in Drosophila melanogaster
Source: PLoS Genet. 2015 Sep 21;11(9):e1005523. doi: 10.1371/journal.pgen.1005523 (PMC4577213; doi:10.1371/journal.pgen.1005523)

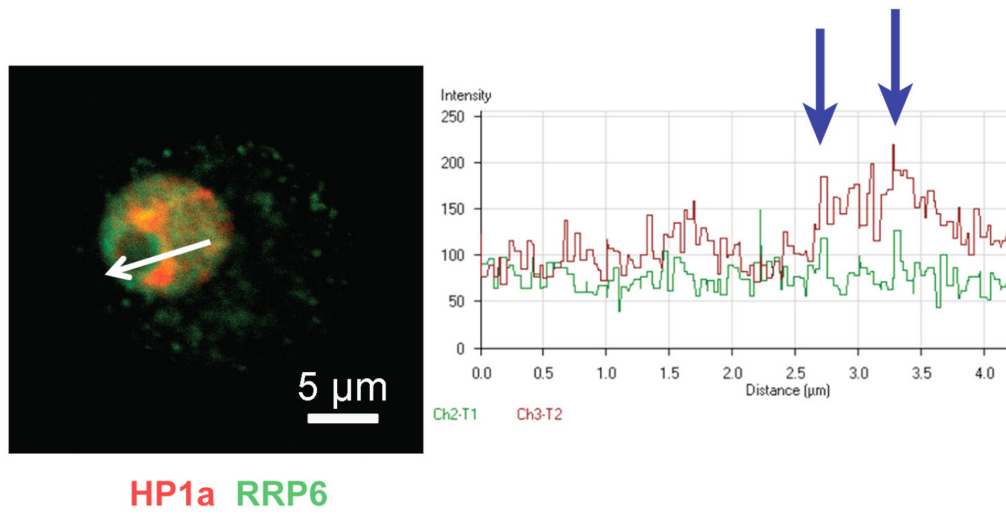

**Figure S1**

Supplement: S1 Fig — Immunofluorescent staining of fixed S2 cells with antibodies against RRP6 (green) and HP1a (red). The fluorescence profile in the right part of the image shows the co-variation of the fluorescent signals in each channel along an axis through the nucleus (white arrow). The distributions of RRP6 and HP1a are different, but both proteins colocalize in some regions of the nucleus. The blue arrows show regions of co-localization. (PDF) [file pgen.1005523.s001.pdf]

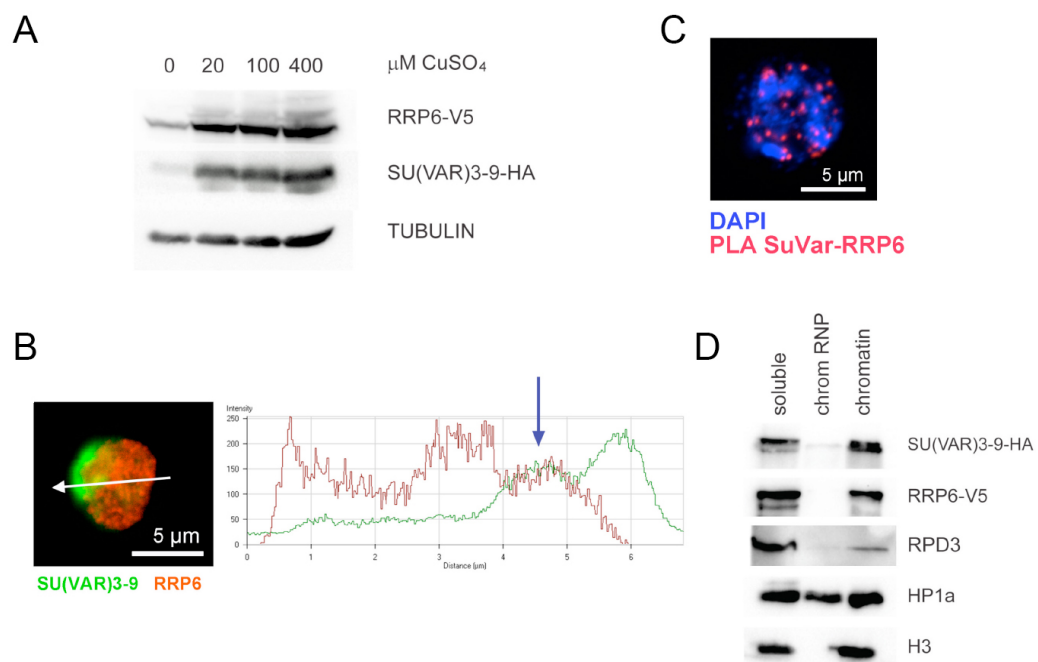

**Figure S2**

Supplement: S2 Fig — (A) Simultaneous expression of V5-tagged RRP6 and HA-tagged SU(VAR)3-9 in S2 cells. S2 cells stably transfected with plasmids for expression of RRP6-V5 and the SU(VAR)3-9-HA (see Materials and Methods for details) were induced with different concentrations of CuSO4, and the expressions of the RRP6-V5 and the SU(VAR)3-9-HA proteins were detected by Western blotting using the anti-V5 antibody and the anti-HA antibody, respectively. Tubulin served as a loading control. (B) Colocalization of SU(VAR)3-9 and RRP6 in Drosophila S2 cells. Immunofluorescent staining of S2 cells that expressed HA-tagged SU(VAR)3-9 and V5-tagged RRP6. The cells were fixed and stained with antibodies against HA (green) and V5 (red). The fluorescence profile in the right part of the image shows the co-variation of the fluorescent signals in each channel along an axis through the nucleus (white arrow). The distributions of RRP6 and SU(VAR)3-9 are different, but both proteins colocalize in some regions of the nucleus (blue arrow). (C) Proximity ligation assay (PLA) showing close proximity between SU(VAR)3-9 and RRP6 in Drosophila S2 cells. S2 cells that expressed HA-tagged SU(VAR)3-9 and V5-tagged RRP6 were double stained with antibodies against HA and V5, and the proximity was assayed using DuoLink probes (red signal). The cells were counterstained with DAPI (blue). (D) Nuclear fraction analysis of the Rrp6—Su(var)3-9 cells. Protein expression in the Rrp6—Su(var)3-9 cells was induced with 200 μM CuSO4 overnight. The cells were harvested and the nuclei were isolated as described in Materials and Methods. The nuclei were separated into soluble (nucleoplasm), chromosomal RNP, and chromatin fractions according to the scheme shown in Fig 2A. The different fractions were analyzed by SDS-PAGE and Western blotting. (PDF) [file pgen.1005523.s002.pdf]

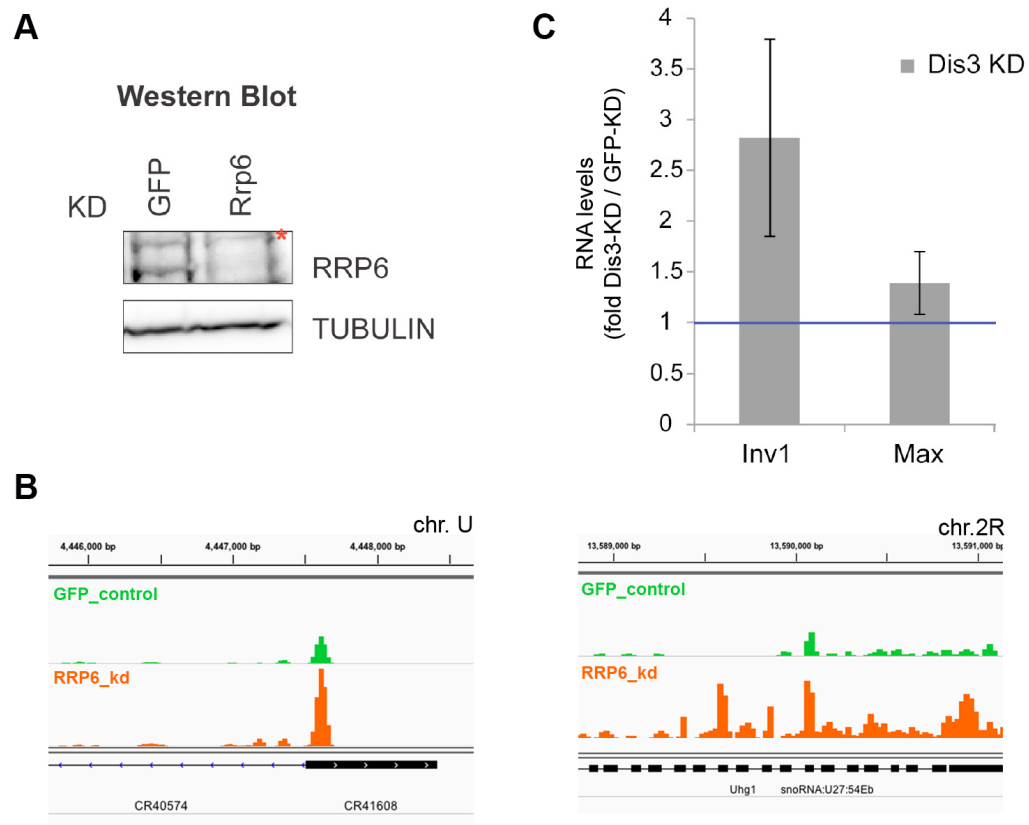

**Figure S3**

Supplement: S3 Fig — (A) Analysis of RRP6 knockdown efficiency. S2 cells were treated with long dsRNA against Rrp6, or against GFP as a control, to deplete the cells of RRP6 protein (see Supplementary Materials and Methods, S1 Text, for details). The cells were harvested 96 hours after the first dsRNA administration. The efficiencies of the knockdown treatments were determined by SDS-PAGE and Western blotting using an antibody against RRP6. Tubulin served as a loading control. The red asterisk in the figure indicates a background signal of the antibody. (B) RRP6 depletion resulted in pre-rRNA processing defects. RRP6 depletion inhibits the trimming of the 3' end of the pre-rRNA CR41608, as shown by the increased amount of RNA complementary to the 3' end of the gene (left panel). RRP6 is also needed for the processing of other functional RNAs, including snoRNAs, and depletion of RRP6 leads to increased levels of snoRNA transcripts (right panel). (C) Depletion of DIS3 resulted in increased levels of some heterochromatic transcripts. In S2 cells depleted of DIS3 (and GFP as control), the RNA levels of two selected heterochromatic transposon sequences (Inv1 and Max) were measured by RT-qPCR. The data from DIS3-KD cells and GFP control cells were normalized to Actin 5C and the results are expressed as a fold change comparing the levels obtained in the DIS3-KD with the levels in the GFP control (the blue line indicates no change). The bars represent averages and the error bars standard deviations from three independent biological replicates. (PDF) [file pgen.1005523.s003.pdf]

**A**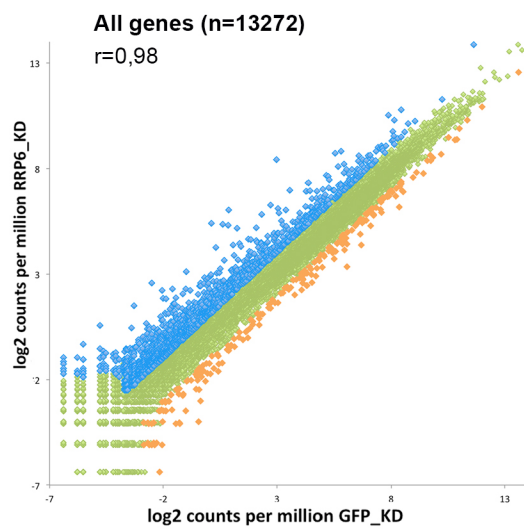**B**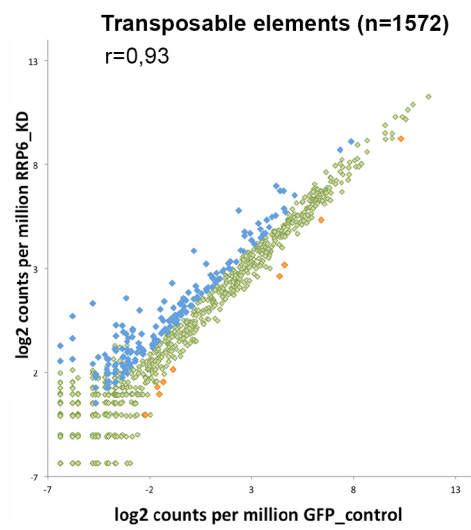**Figure S4**

Supplement: S4 Fig — S2 cells were treated with long dsRNA against Rrp6 to deplete the cells of RRP6 protein, or against GFP as a control, as in S3 Fig. Expression levels were analysed by RNA-seq and the figure shows comparisons between knockdown of RRP6 and the GFP control samples. (A) All ORF and ncRNAs (n = 13272). 1534 genes showed increased expression levels (average log2 ratio > 1, blue). 213 genes showed decreased expression levels (average log2 ratio < 1, orange). (B) Transposable elements (n = 1572). 75 transposons showed increased expression levels (average log2 ratio > 1, blue). 9 transposons showed decreased expression levels (average log2 ratio < 1, orange). r indicates Pearson’s correlation coefficient. (PDF) [file pgen.1005523.s004.pdf]

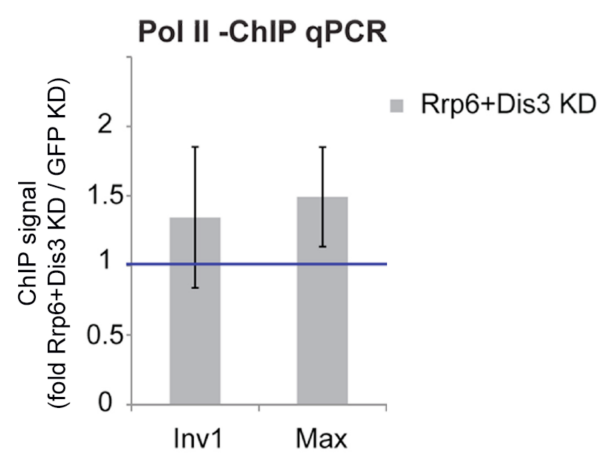

**Figure S6**

Supplement: S6 Fig — ChIP experiments with an antibody against RNA polymerase II in S2 cells depleted of RRP6 and DIS3. ChIP signals were calculated relative to the corresponding input sample. An external standard was used for normalization as described by Eberle et al. (2012) [54]. The histogram shows the average signals and standard deviations of the fold change obtained when comparing the ChIP signals in the Rrp6+Dis3 KD with those in the control GFP KD. Data from four independent biological replicates. (PDF) [file pgen.1005523.s006.pdf]

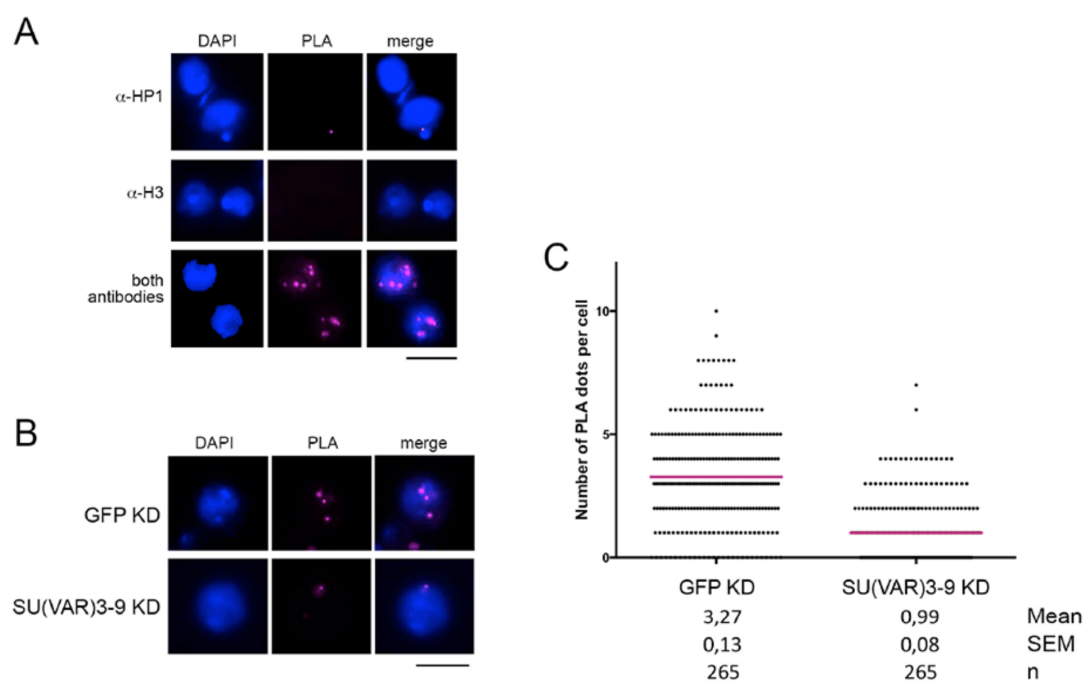

**Figure S7**

Supplement: S7 Fig — S2 cells were analysed by PLA with antibodies against HP1a and histone H3. The rationale of the assay is that a more open chromatin conformation reduces the interaction between HP1a and histone H3. (A) PLA analysis of control S2 cells. The slides were counterstained with DAPI (blue). The figure shows negative control reaction with each of the antibodies separately. PLA signals (magenta dots) were observed only in the presence of both antibodies and were restricted to the cell nucleus, as expected. (B) PLA analysis of cells depleted of SU(VAR)3-9. A control experiment was carried out to assess the suitability of the H3-HP1a PLA assay to detect changes in chromatin compaction. The number of PLA dots per cells in cells depleted of SU(VAR)3-9 was compared to that observed in GFP control cells. The figure shows representative examples of the results obtained in each condition. (C) Quantitative analysis of the results of the experiment described in B. The graph shows the results obtained from 265 cells analysed in each condition. Depletion of SU(VAR)3-9 reduced more than three-fold the number of PLA dots per cell. This difference was highly significant (P<0.0001 in a two-tailed, nonparametric Mann Whitney test). The magenta bars in the graph indicated the mean value in each condition. Mean and SEM values for each condition are provided below the graph. (PDF) [file pgen.1005523.s007.pdf]

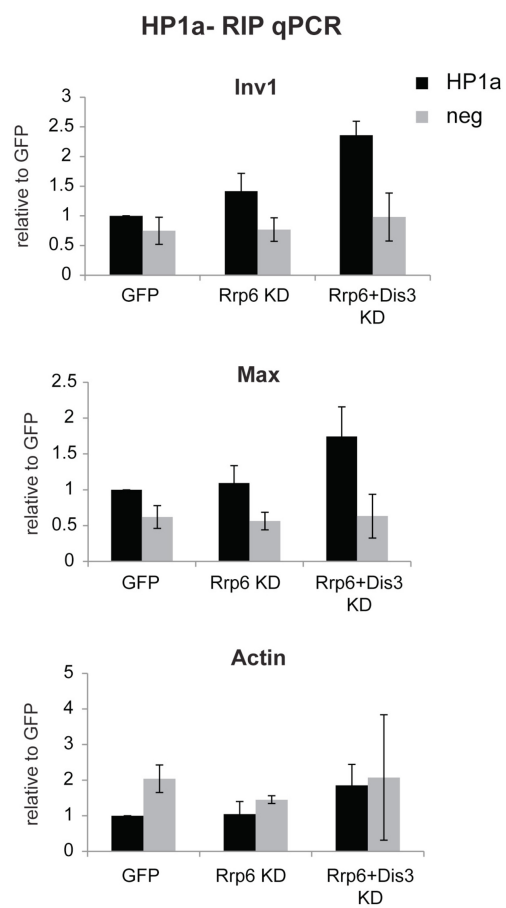

**Figure S8**

Supplement: S8 Fig — RIP experiments were performed with an anti-HP1a antibody (black bars) and no-antibody controls (neg; grey bars) (for details see Materials and Methods). The immunoprecipitated RNA was reverse transcribed into cDNA and specific sequences (Inv1, Max, and Actin) were analyzed by RT-qPCR. The data was set relative to the GFP sample. The figure shows averages and standard deviations obtained from two independent biological replicates, each quantified in duplicate. HP1a bound to RNA in the chromatin is undetectable in the GFP controls (RIP signals for HP1a are background levels), but a significant increase was measured in cells depleted of exosome ribonucleases (RIP signals above background levels). (PDF) [file pgen.1005523.s008.pdf]

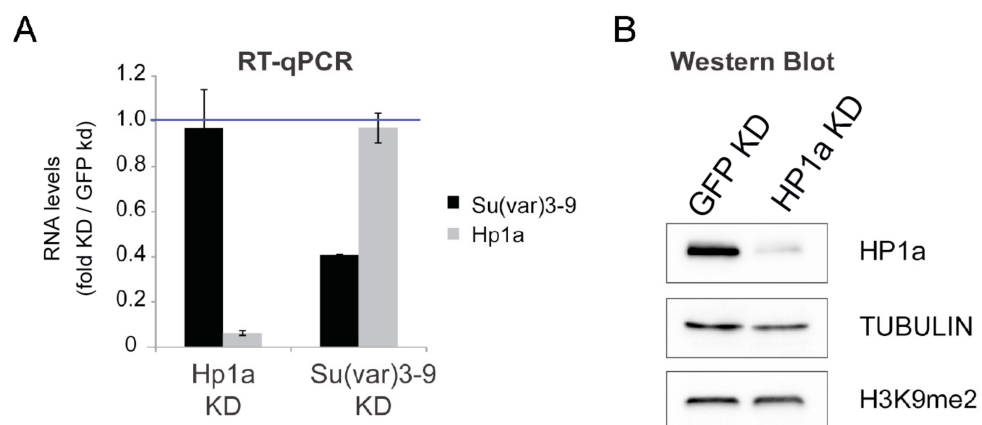

**Figure S9**

Supplement: S9 Fig — (A) S2 cells were treated with dsRNA against GFP (control), Hp1a, or Su(var)3-9. To analyze the knockdown efficiencies, total RNAs were purified and were reverse transcribed into cDNA, and the resulting cDNAs were analyzed by qPCR. The RNA levels were normalized to Actin 5C and expressed as a fold change compared to the GFP control. Averages and standard deviations of three (for Hp1a) and two (for Su(var)3-9) independent experiments are shown in the figure. (B) The Hp1a knockdown was also determined by Western blotting. Tubulin and H3K9me2 served as loading controls. (PDF) [file pgen.1005523.s009.pdf]

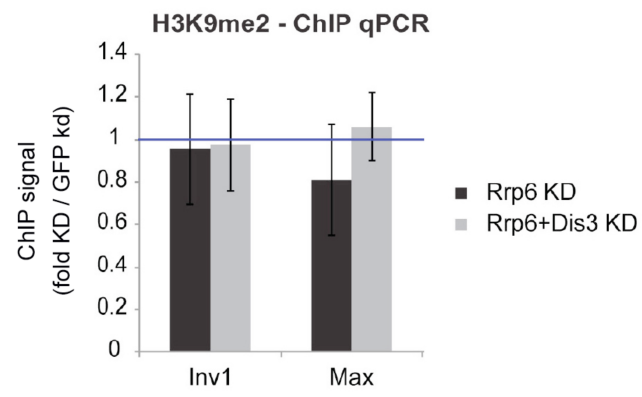

**Figure S10**

Supplement: S10 Fig — Depletion of RRP6 and DIS3 did not cause any significant change in the levels of H3K9me2 at the analysed sites. Chromatin was harvested from S2 cells depleted of GFP (as a control), RRP6, or RRP6 and DIS3 together. ChIP experiments were performed with the anti-H3K9me2 antibody. The ChIP signals were calculated relative to the corresponding input and normalized to an external reference DNA sequence that was added in the beginning of the experiment (see Eberle et al., 2012) [54]. The histogram shows the average signals and standard deviations expressed as a fold change compared to the GFP control from four independent biological replicates for RRP6 KD (black bars) and three for the double knockdown (grey bars), each with one qPCR run. (PDF) [file pgen.1005523.s010.pdf]

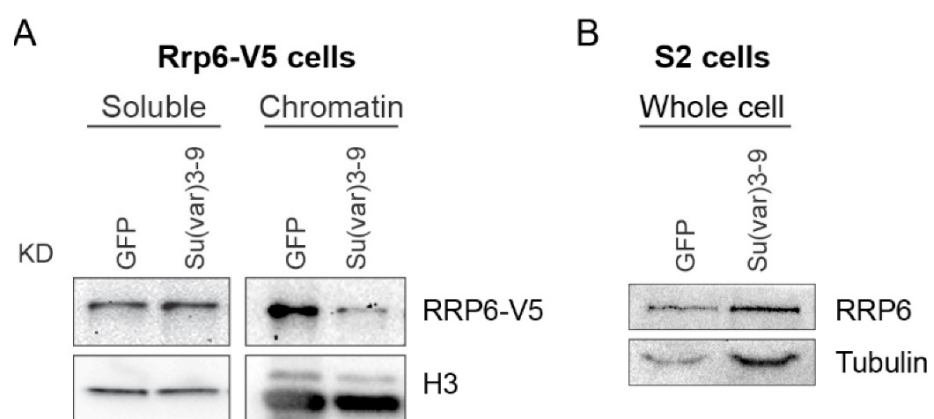

**Figure S11**

Supplement: S11 Fig — (A) S2-RRP6-V5 cells were treated with dsRNA against GFP (control) or Su(var)3-9. Nuclei were isolated and fractionated, and the soluble and chromatin fractions were analysed by Western blotting. Reduced RRP6 signal was observed in the chromatin pellet but not in the soluble fraction, which suggests that the overall levels of expression of RRP6 are not reduced upon Su(Var)3-9 KD. (B) S2 cells were treated with dsRNA against GFP (control) or Su(var)3-9. Whole-cell extracts were prepared and analyzed by Western blotting. (PDF) [file pgen.1005523.s011.pdf]

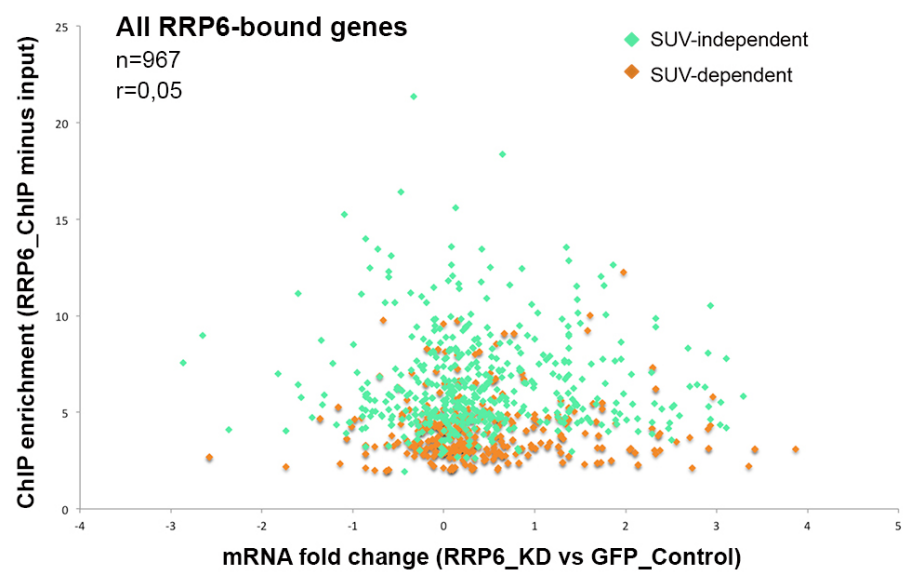

**Figure S13**

Supplement: S13 Fig — Scatter plot showing the levels of RRP6-occupancy in S2 cells (measured by ChIP-seq in control GFP cells) and the effect of RRP6 depletion (measured by RNA-seq in cells treated with RRP6-dsRNA compared to control GFP cells) for each transcript. Transcripts from SUV-dependent and SUV-independent genes are represented in different colors, as indicated. The Spearman’s rank correlation coefficient is indicated (r). (PDF) [file pgen.1005523.s013.pdf]

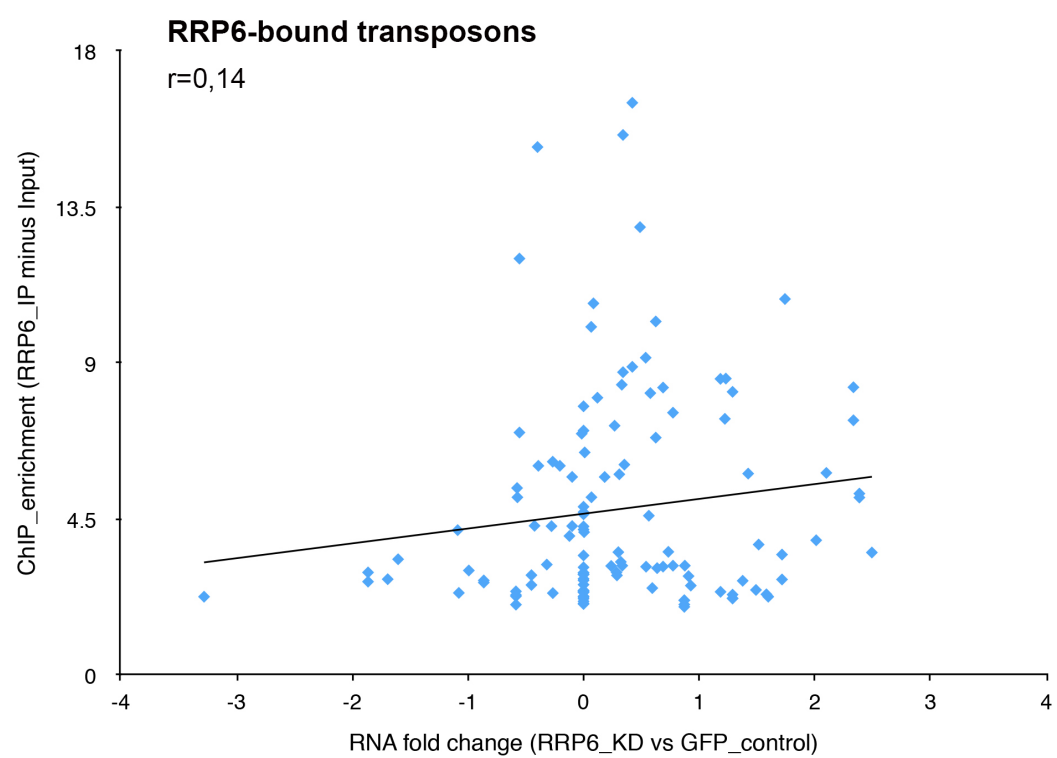

**Figure S14**

Supplement: S14 Fig — Scatter plot showing the levels of RRP6-occupancy in S2 cells (measured by ChIP-seq in control GFP cells) and the effect of RRP6 depletion (measured by RNA-seq in cells treated with RRP6-dsRNA compared to control GFP cells) for each transposon. The Spearman’s rank correlation coefficient is indicated (r). (PDF) [file pgen.1005523.s014.pdf]

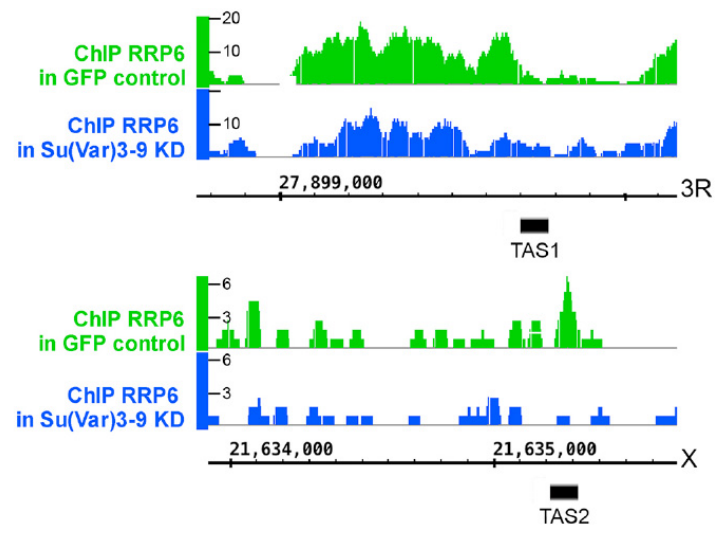

Figure S15

Supplement: S15 Fig — ChIP-seq experiments were carried out using S2 cells that expressed V5-tagged RRP6 under low-induction conditions, as in S8 Fig. The expression of Su(Var)3-9 was knocked down using dsRNA. Control cells were treated in parallel with GFP-dsRNA. The image shows examples of RRP6 occupancy in two selected genomic regions that are known to generate piRNAs during early development (Yin & Lin, 2007; Nature 450:304–308). The data was visualized and the images generated using the Integrated Genome Browser v. 8.1.11. (PDF) [file pgen.1005523.s015.pdf]
