# Supplementary material for: An Interaction between RRP6 and SU(VAR)3-9 Targets RRP6 to Heterochromatin and Contributes to Heterochromatin Maintenance in Drosophila melanogaster
Source: PLoS Genet. 2015 Sep 21;11(9):e1005523. doi: 10.1371/journal.pgen.1005523 (PMC4577213; doi:10.1371/journal.pgen.1005523)
Supplement: S5 Fig — (A) Analysis of genomic DNA contamination in cDNA samples. RNA samples (total RNA and chromatin-associated RNA) were treated with DNAse and reverse transcribed into cDNA as described in the Materials and Methods. RT (-) control reactions were processed in parallel without adding reverse transcriptase to the RT reaction mixture. The Actin 5C levels in RT(+) and RT(-) samples were analyzed by RT-qPCR and compared to each other to quantify possible genomic DNA contamination in the cDNA samples. The table shows data from three independent experiments (EXP I, II, III). The figures are the percentages of genomic contamination in the cDNA samples. (B) MNase experiments showed nucleosome density on analyzed regions of the genome. Chromatin prepared from S2 cells was treated with 20 U MNase or with no MNase (for details see the Materials and Methods). After DNA purification, the MNase-treated samples were compared to untreated samples by qPCR. A low value in a specific genomic region means that this region is nucleosome-free (or less condensed), while a high value in a genomic region points towards a more compact chromatin structure. Hsp70 was used as a control since the promoter is nucleosome-free and a positioned nucleosome is present in the coding region [39]. The figure shows averages and standard deviations from three independent experiments. These control experiments based on the analysis of the Hsp70 promoter showed that open chromatin regions were more accessible to MNase than compact regions, and are therefore less represented in the digested chromatin preparations. These experiments also showed that the three analyzed heterochromatic regions were approximately four times more represented than the open Hsp70 used as a control, which is consistent with the heterochromatic nature of these regions. (PDF) [file pgen.1005523.s005.pdf]

A

% DNA contamination
(measured for Actin)

	EXP I	EXP II	EXP III
**total RNA**	0.0026	0.0064	0.00279
**chromatin associated RNA**	0.0043	0.0076	0.00093

B

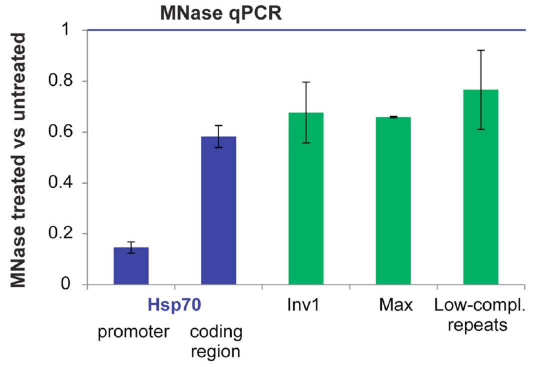

Figure S5
