# Supplementary material for: An Interaction between RRP6 and SU(VAR)3-9 Targets RRP6 to Heterochromatin and Contributes to Heterochromatin Maintenance in Drosophila melanogaster
Source: PLoS Genet. 2015 Sep 21;11(9):e1005523. doi: 10.1371/journal.pgen.1005523 (PMC4577213; doi:10.1371/journal.pgen.1005523)
Supplement: S12 Fig — ChIP-seq experiments were carried out using S2 cells that expressed V5-tagged RRP6 under low-induction conditions. The expression of Su(Var)3-9 was knocked down using dsRNA. Control cells were treated in parallel with GFP-dsRNA. (A) ChIP-seq parameters. The table indicates the number of reads mapped to the genome, the number of RRP6-rich regions determined by MACS version 2.1.0 with a q-value cutoff of 5.00e-02, the average length and fold enrichment of the peaks in each condition. (B) The meta-gene distribution of RRP6 computed by MACS2, including 1 kb upstream of the transcription-start site (TSS) and 1 kb downstream of the transcription-termination site (TTS). (C) Upregulated and downregulated RRP6-rich regions in the different chromosomes of D. melanogaster. The table also shows the chromosome distribution of RRP6-rich regions in control, GFP-treated cells. Upregulated RRP6-rich regions are regions that are found in cells depleted of Su(Var)3-9 but not in GFP control cells. Downregulated regions are those that are present in GFP control cells but not in Su(Var)3-9-depleted cells. Peaks with at least 50% length overlap were considered to be the same peak and were not considered changed in this analysis. (D) Examples of RRP6 occupancy in selected loci corresponding to different types of sequences, as indicated in the figure. The data was visualized and the images generated using the Integrated Genome Browser v. 8.1.11. (PDF) [file pgen.1005523.s012.pdf]

A

Treatment	Sample	Nr. mapped reads	Nr. RRP6-rich regions	q-value cutoff	Average length (bp)	Average fold enrichment
GFP control	Input DNA	10,525,698				
Su(Var)3-9-KD	Input DNA	9,962,593				
GFP control	ChIP DNA	12,190,050	5447	5.00e-02	134,9	4,7
Su(Var)3-9-KD	ChIP DNA	8,191,052	4733	5.00e-02	120,5	5,5

B

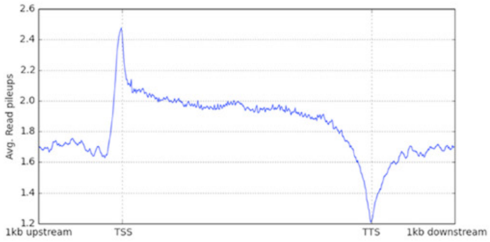

C

Chromosome	Nr. RRP6-rich regions in GFP control	Nr. regions downregulated in Su(Var)3-9 KD	Nr. regions upregulated in Su(Var)3-9 KD
X	928	146	151
2L	399	138	103
2R	424	137	74
3L	390	145	105
3R	582	166	134
4	5	5	6
U	132	77	20
Uextra	2505	683	220
XHet	4	0	1
YHet	4	3	1
3RHet	32	22	1
3LHet	16	12	1
2RHet	23	17	6
2LHet	3	2	0

D

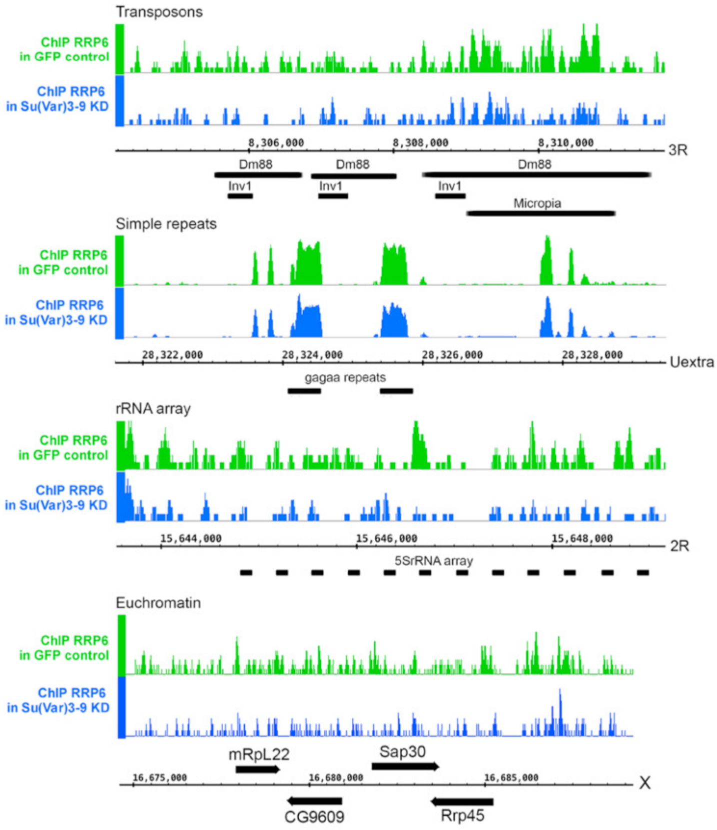

Figure S12
