# Supplementary material for: An Interaction between RRP6 and SU(VAR)3-9 Targets RRP6 to Heterochromatin and Contributes to Heterochromatin Maintenance in Drosophila melanogaster
Source: PLoS Genet. 2015 Sep 21;11(9):e1005523. doi: 10.1371/journal.pgen.1005523 (PMC4577213; doi:10.1371/journal.pgen.1005523)
Supplement: S1 Table — (PDF) [file pgen.1005523.s016.pdf]

Table S1. List of Rrp6 interactors (FDR < 0.01)

GI	Gene_Name*	P-value	Q-value	Log-fold-enrichment
45549251	misshapen CG16973-PA, isoform A	1E-15	8,71E-17	7,81
13560842	heat shock protein Hsp70Ba	1E-15	8,71E-17	7,79
24653408	Myb-interacting protein 120 CG6061-PA	1E-15	8,71E-17	7,03
17933682	small bristles CG1664-PA	1E-15	8,71E-17	6,90
71490	heat shock protein 26 - fruit fly	1E-15	8,71E-17	6,45
16265798	nucleosome remodeling factor large subunit NURF301	1E-15	8,71E-17	6,34
24647320	serpent CG3992-PB, isoform B	1E-15	8,71E-17	5,94
6434958	26S proteasome regulatory complex subunit p42D	1E-15	8,71E-17	5,88
28574115	CG4747-PA	1E-15	8,71E-17	5,81
24646242	MBD-R2 CG10042-PA, isoform A	1E-15	8,71E-17	5,75
17137252	viking CG16858-PA	1E-15	8,71E-17	5,70
24647046	Suppressor of variegation 3-9 CG6476-PB, isoform B	1E-15	8,71E-17	5,69
24658883	CG3800-PA	1E-15	8,71E-17	5,66
24645257	CG11033-PA	1E-15	8,71E-17	5,51
600424	FKBP39	1E-15	8,71E-17	5,46
17737397	Kruppel homolog 2 CG9159-PA	1E-15	8,71E-17	5,35
24652438	midlife-crisis CG11763-PA, isoform A	1E-15	8,71E-17	5,29
45550973	Phosphoethanolamine cytidylyltransferase CG5547-PB	1E-15	8,71E-17	5,09
157906	maleless protein	1E-15	8,71E-17	5,04
7833	dynamin	1E-15	8,71E-17	5,01
24665814	CG6512-PB, isoform B	1E-15	8,71E-17	4,99
24640727	Bx42 CG8264-PA	1E-15	8,71E-17	4,94
24640482	female sterile (1) homoeotic CG2252-PB, isoform B	1E-15	8,71E-17	4,91
1170808	Longitudinals lacking protein, isoform G	1E-15	8,71E-17	4,88
24639372	CG8636-PA	1E-15	8,71E-17	4,86
18921137	Ribosomal protein L17 CG3203-PD, isoform D	1E-15	8,71E-17	4,83
24583463	Fatty acid (long chain) transport protein CG7400-PA	1E-15	8,71E-17	4,81
21392078	RE09547p	1E-15	8,71E-17	4,80
3676167	coatomer alpha subunit	1E-15	8,71E-17	4,79
18860081	Yippee CG1989-PA, isoform A	1E-15	8,71E-17	4,77
19921856	CG8230-PA	1E-15	8,71E-17	4,77
18859989	CG9281-PB, isoform B	1E-15	8,71E-17	4,74
24583731	crooked legs CG14938-PD, isoform D	1E-15	8,71E-17	4,70
78706838	Muscle-specific protein 300 CG33715-PD, isoform D	1E-15	8,71E-17	4,68
606751	RNA binding protein	1E-15	8,71E-17	4,65
17136888	RNA polymerase II elongation factor CG3710-PA	1E-15	8,71E-17	4,65
24660036	sec63 CG8583-PA	1E-15	8,71E-17	4,60

21356859	Rpn1 CG7762-PA	1E-15	8,71E-17	4,56
28573444	Rrp42 CG8395-PA	1E-15	8,71E-17	4,52
17862648	LD43057p	1E-15	8,71E-17	4,50
1401254	nucleoplasmin-like protein-short	1E-15	8,71E-17	4,45
24639109	CG14782-PA	1E-15	8,71E-17	4,44
19922030	Translocon-associated protein delta CG9035-PA	1E-15	8,71E-17	4,44
18860035	Dlic2 CG1938-PA, isoform A	1E-15	8,71E-17	4,43
21355403	CG4452-PA, isoform A	1E-15	8,71E-17	4,43
20130427	CG2199-PA, isoform A	1E-15	8,71E-17	4,43
21356927	Ribosomal protein L35A CG2099-PA	1E-15	8,71E-17	4,39
28573655	pita CG3941-PA, isoform A	1E-15	8,71E-17	4,38
17864298	hoi-polloi CG3949-PA	1E-15	8,71E-17	4,36
16648488	SD05406p	1E-15	8,71E-17	4,35
17647893	Ribosomal protein S26 CG10305-PB, isoform B	1E-15	8,71E-17	4,34
85816181	hangover CG32575-PB, isoform B	1E-15	8,71E-17	4,33
24646936	Rrp6 CG7292-PA	1E-15	8,71E-17	4,33
17137634	smt3 CG4494-PA	1E-15	8,71E-17	4,32
21430048	GM13065p	1E-15	8,71E-17	4,32
20129993	CG10131-PA	1E-15	8,71E-17	4,32
126746	205 kDa microtubule-associated protein	1E-15	8,71E-17	4,31
21357009	Ribosomal protein L18 CG8615-PA	1E-15	8,71E-17	4,24
45549187	Nucleosome remodeling factor - 38kD CG4634-PA	1E-15	8,71E-17	4,22
17647879	Ribosomal protein L13 CG4651-PA, isoform A	1E-15	8,71E-17	4,21
17933574	cut up CG6998-PA, isoform A	1E-15	8,71E-17	4,20
24642563	Nup153 CG4453-PA	1E-15	8,71E-17	4,19
158749	tubulin-beta-3	1E-15	8,71E-17	4,19
21357575	CTP phosphocholine cytidylyltransferase 1 CG1049-PA	1E-15	8,71E-17	4,17
33589646	AT24152p	1E-15	8,71E-17	4,17
7919	unnamed protein product	1E-15	8,71E-17	4,17
2706522	ubiquitin activating enzyme	1E-15	8,71E-17	4,16
6491997	SPT6 protein	1E-15	8,71E-17	4,14
8444	unnamed protein product	1E-15	8,71E-17	4,11
4378008	ribosomal protein L22	1E-15	8,71E-17	4,09
21355245	CG6322-PA	1E-15	8,71E-17	4,09
17975579	Ribosomal protein S14a CG1524-PB, isoform B	1E-15	8,71E-17	4,06
24641266	CG1737-PA	1E-15	8,71E-17	4,06
10334685	phosphoglycerate mutase	1E-15	8,71E-17	4,05
6093992	60S ribosomal protein L10 (QM protein homolog) (dQM)	1E-15	8,71E-17	4,02
17647889	Ribosomal protein S17 CG3922-PB	1E-15	8,71E-17	4,01
19922464	eIF3-S8 CG4954-PA	1E-15	8,71E-17	3,99
17137198	Ribosomal protein L9 CG6141-PA, isoform A	1E-15	8,71E-17	3,97
3953611	MCM7	1E-15	8,71E-17	3,97
17864162	Ribosomal protein S3A CG2168-PA, isoform A	1E-15	8,71E-17	3,96
18859933	lethal (1) G0022 CG8231-PA	1E-15	8,71E-17	3,94
19922746	Ribosomal protein S16 CG4046-PA	1E-15	8,71E-17	3,93

17648041	Trip1 CG8882-PA	1E-15	8,71E-17	3,93
24583646	CG4738-PA	1E-15	8,71E-17	3,92
24657454	CG1316-PA	1E-15	8,71E-17	3,90
24664062	CG32138-PA, isoform A	1E-15	8,71E-17	3,88
16185578	LD42783p	1E-15	8,71E-17	3,87
24762707	CG4806-PA	1E-15	8,71E-17	3,85
103304	phosphoprotein phosphatase (EC 3.1.3.16) 65K regulatory chain - fruit fly	1E-15	8,71E-17	3,84
17157991	thioredoxin peroxidase 1 CG1633-PA, isoform A	1E-15	8,71E-17	3,83
24655737	beta-Tubulin at 56D CG9277-PB, isoform B	1E-15	8,71E-17	3,81
19921802	CG2158-PA	1E-15	8,71E-17	3,81
17136574	Ribosomal protein S27A CG5271-PA	1E-15	8,71E-17	3,80
17137572	Elongation factor 1alpha48D CG8280-PA, isoform A	1E-15	8,71E-17	3,79
28571526	CG31549-PA	1E-15	8,71E-17	3,79
24645909	CG5214-PA	1E-15	8,71E-17	3,77
17738165	Heat shock protein 68 CG5436-PA	1E-15	8,71E-17	3,75
17737967	Heat shock protein cognate 4 CG4264-PA, isoform A	1E-15	8,71E-17	3,75
19921654	CG10417-PA, isoform A	1E-15	8,71E-17	3,74
19921922	Ribosomal protein L31 CG1821-PB, isoform B	1E-15	8,71E-17	3,74
24585720	CG1416-PA, isoform A	1E-15	8,71E-17	3,73
24665395	CG4169-PA	1E-15	8,71E-17	3,70
17647521	Heat shock protein 27 CG4466-PA	1E-15	8,71E-17	3,70
19528335	LD21358p	1E-15	8,71E-17	3,66
24656161	CG1244-PA, isoform A	1E-15	8,71E-17	3,64
19922004	Rpb5 CG11979-PA	1E-15	8,71E-17	3,64
17738077	Ribosomal protein S20 CG15693-PA	1E-15	8,71E-17	3,63
1666637	histone deacetylase	1E-15	8,71E-17	3,62
1168016	26S protease regulatory complex non-ATPase subunit	1E-15	8,71E-17	3,58
19921810	CG8707-PA	1E-15	8,71E-17	3,57
6433838	DNop5 protein	1E-15	8,71E-17	3,57
17864148	gamma-coatomer protein CG1528-PA, isoform A	1E-15	8,71E-17	3,57
6942155	putative cytoplasmic aminopeptidase	1E-15	8,71E-17	3,57
17945866	RE39378p	1E-15	8,71E-17	3,56
17647553	ken and barbie CG5575-PA	1E-15	8,71E-17	3,55
21356869	Su(z)12 CG8013-PA, isoform A	1E-15	8,71E-17	3,54
16197833	GH10480p	1E-15	8,71E-17	3,54
21428348	GM02257p	1E-15	8,71E-17	3,51
17647885	Ribosomal protein L18A CG6510-PA	1E-15	8,71E-17	3,51
1072120	nucleosome assembly protein NAP-1	1E-15	8,71E-17	3,50
21355533	CG8108-PB, isoform B	1E-15	8,71E-17	3,49
78707302	kayak CG33956-PD, isoform D	1E-15	8,71E-17	3,49
45549221	Suppressor of variegation 3-7 CG8599-PA	1E-15	8,71E-17	3,49
24649976	Ribosomal protein S27 CG10423-PA	1E-15	8,71E-17	3,47
6631000	bonus	1E-15	8,71E-17	3,46
18860045	Klp10A CG1453-PA, isoform A	1E-15	8,71E-17	3,46
54650868	GM06787p	1E-15	8,71E-17	3,45
4325130	dMi-2 protein	1E-15	8,71E-17	3,45
16768870	LD09503p	1E-15	8,71E-17	3,39

157810	laminin receptor	1E-15	8,71E-17	3,38
1305408	D-stat protein short form	1E-15	8,71E-17	3,38
24584738	CG31739-PA	1E-15	8,71E-17	3,38
24658349	CG10630-PA	1E-15	8,71E-17	3,37
17136324	Ribosomal protein S3 CG6779-PA	1E-15	8,71E-17	3,37
17136986	twinstar CG4254-PA	1E-15	8,71E-17	3,37
21358013	CG1957-PA, isoform A	1E-15	8,71E-17	3,36
17137796	G protein salpha 60A CG2835-PB, isoform B	1E-15	8,71E-17	3,35
24653714	Boundary element-associated factor of 32kD CG10159-PB	1E-15	8,71E-17	3,35
62471723	CG33505-PA	1E-15	8,71E-17	3,35
8356	protein kinase	1E-15	8,71E-17	3,34
17737290	Ribosomal protein S6 CG10944-PB, isoform B	1E-15	8,71E-17	3,34
17975567	Ribosomal protein S15Aa CG2033-PD, isoform D	1E-15	8,71E-17	3,33
16768790	LD03515p	1E-15	8,71E-17	3,33
19922556	CG5482-PA	1E-15	8,71E-17	3,33
2950374	C-terminal binding protein	1E-15	8,71E-17	3,30
17737463	Imitation SWI CG8625-PA, isoform A	1E-15	8,71E-17	3,30
17647581	lark CG8597-PA, isoform A	1E-15	8,71E-17	3,30
2947310	nucleoporin	1E-15	8,71E-17	3,29
158200	Dras1 protein	1E-15	8,71E-17	3,29
13446610	putative exoribonuclease DIS3	1E-15	8,71E-17	3,29
20129705	Ribosomal protein L21 CG12775-PA	1E-15	8,71E-17	3,29
17137740	Proteasome p44.5 subunit CG10149-PB, isoform B	1E-15	8,71E-17	3,28
18860087	eIF-2alpha CG9946-PA	1E-15	8,71E-17	3,28
19923002	CG12030-PA	1E-15	8,71E-17	3,28
17737731	Ribosomal protein LP0 CG7490-PA	1E-15	8,71E-17	3,27
19921464	CG6453-PA	1E-15	8,71E-17	3,24
24647704	Splicing factor 1 CG5836-PA	1E-15	8,71E-17	3,23
17946442	RE67757p	1E-15	8,71E-17	3,23
158821	zinc-finger homeodomain protein 1	1E-15	8,71E-17	3,22
11024346	heat shock protein Hsp70Aa	1E-15	8,71E-17	3,22
21355591	Srp68 CG5064-PA	1E-15	8,71E-17	3,22
22024141	Ribosomal protein S23 CG8415-PA	1E-15	8,71E-17	3,20
2290597	RACK1	1E-15	8,71E-17	3,19
158769	ubiquitin	1E-15	8,71E-17	3,18
24643970	CG14648-PB, isoform B	1E-15	8,71E-17	3,17
24584645	CG31738-PB, isoform B	1E-15	8,71E-17	3,15
24586291	Cyt-b5 CG2140-PB, isoform B	1E-15	8,71E-17	3,14
17136564	alpha-Tubulin at 84B CG1913-PA	1E-15	8,71E-17	3,11
24643266	CG14200-PA	1E-15	8,71E-17	3,11
8488	unnamed protein product	1E-15	8,71E-17	3,10
28571872	baiser CG11785-PA	1E-15	8,71E-17	3,09
24586038	CG17266-PA	1E-15	8,71E-17	3,09
17865835	Rab-protein 7 CG5915-PA	1E-15	8,71E-17	3,08
17736973	Rab-protein 5 CG3664-PE, isoform E	1E-15	8,71E-17	3,06
21358459	Ribosomal protein S7 CG1883-PA, isoform A	1E-15	8,71E-17	3,05
45550830	Nup358 CG11856-PA	1E-15	8,71E-17	3,05
3309275	karyopherin alpha 3	1E-15	8,71E-17	3,04
24649978	CG11875-PA	1E-15	8,71E-17	3,03

21357547	CG8863-PA, isoform A	1E-15	8,71E-17	3,01
24668543	Isoleucyl-tRNA synthetase CG11471-PA, isoform A	1E-15	8,71E-17	3,01
156750	actin	1E-15	8,71E-17	3,01
24651986	Suppressor of variegation 2-10 CG8068-PI, isoform I	1E-15	8,71E-17	2,99
19921728	CG11107-PA	1E-15	8,71E-17	2,98
25012530	RE33426p	1E-15	8,71E-17	2,97
558485	ribosomal protein DL11	1E-15	8,71E-17	2,97
19921434	CaBP1 CG5809-PA	1E-15	8,71E-17	2,92
17647529	Heat shock protein 83 CG1242-PA	1E-15	8,71E-17	2,91
24639740	lethal (1) G0334 CG7010-PC, isoform C	1E-15	8,71E-17	2,90
440853	ribosomal protein S4	1E-15	8,71E-17	2,89
21355785	CG8436-PA	1E-15	8,71E-17	2,88
2209280	SLY1 homologous	1E-15	8,71E-17	2,87
25012218	LD02622p	1E-15	8,71E-17	2,87
20128923	CG2918-PA	1E-15	8,71E-17	2,86
6517192	Drab2	1E-15	8,71E-17	2,85
17737907	Ribosomal protein L3 CG4863-PA, isoform A	1E-15	8,71E-17	2,84
24583962	CG5787-PA	1E-15	8,71E-17	2,82
2245467	DUG	1E-15	8,71E-17	2,80
311343	GTP-binding protein	1E-15	8,71E-17	2,80
1621615	U2 snRNP auxiliary factor	1E-15	8,71E-17	2,76
19920866	CG11266-PB, isoform B	1E-15	8,71E-17	2,76
17975542	Histone H4 replacement CG3379-PC, isoform C	1E-15	9,57E-17	2,74
51091987	SD11791p	1E-15	9,57E-17	2,74
21358145	CG11876-PD, isoform D	1E-15	1,05E-16	2,74
21358615	CG7920-PA, isoform A	1E-15	1,23E-16	2,73
17737957	suppressor of Hairy wing CG8573-PA, isoform A	1E-15	1,51E-16	2,72
1373433	vacuolar ATPase subunit A	1E-15	1,69E-16	2,72
17136268	cropped CG7664-PA	1E-15	1,78E-16	2,72
24649446	Proteasome 26S subunit subunit 4 ATPase CG5289-PA	1E-15	2,14E-16	2,71
20129315	CG5261-PB, isoform B	1E-15	2,41E-16	2,70
7662	Bj6 protein	5,00E-15	4,15E-16	2,68
33088246	adherin Nipped-B	5,55E-15	4,58E-16	2,67
17137592	Int6 homologue CG9677-PA	6,00E-15	4,93E-16	2,67
8484	ribosomal protein	6,11E-15	5,00E-16	2,67
17737553	Heat shock protein 23 CG4463-PA	6,77E-15	5,52E-16	2,67
17647555	Kinesin light chain CG5433-PA	7,99E-15	6,48E-16	2,66
2653645	zinc finger 30C	1,08E-14	8,65E-16	2,65
495594	poly(A)-binding protein	1,24E-14	9,94E-16	2,64
21357495	CG10103-PA	1,51E-14	1,20E-15	2,63
24645384	CG9373-PA	1,81E-14	1,43E-15	2,62
19921458	CG15141-PA	1,91E-14	1,51E-15	2,62
2944333	Na+/K+ ATPase alpha subunit	1,99E-14	1,56E-15	2,62
19921636	cul-2 CG1512-PB, isoform B	2,22E-14	1,74E-15	2,61
24644422	CG10979-PA	2,46E-14	1,92E-15	2,61
4098987	dMyc1	2,59E-14	2,00E-15	2,61
21355207	Spase 22/23-subunit CG5677-PA	2,61E-14	2,01E-15	2,61
2570794	transcriptional co-repressor SIN3A	3,56E-14	2,74E-15	2,59

19920938	CG17293-PA	4,09E-14	3,12E-15	2,59
17647245	T-complex Chaperonin 5 CG8439-PA, isoform A	4,21E-14	3,20E-15	2,59
3108349	pyruvate kinase	4,29E-14	3,25E-15	2,58
24644386	CG2097-PA	6,08E-14	4,59E-15	2,57
24645119	CG8036-PD, isoform D	6,36E-14	4,78E-15	2,57
20129399	Aldehyde dehydrogenase CG3752-PA	8,40E-14	6,29E-15	2,55
385454	SqdA	1,65E-13	1,23E-14	2,52
17864668	belphegor CG6815-PA	1,77E-13	1,32E-14	2,52
19922796	lethal (2) k09913 CG3082-PC, isoform C	2,21E-13	1,63E-14	2,51
17647425	Fibrillarin CG9888-PA	2,23E-13	1,64E-14	2,51
17985987	belle CG9748-PA	2,67E-13	1,96E-14	2,50
21355383	CG9797-PA	3,28E-13	2,40E-14	2,49
7739698	Ran binding protein 7	4,59E-13	3,33E-14	2,48
17647193	beta-coatomer protein CG6223-PA	5,16E-13	3,73E-14	2,47
17737841	Replication Protein A 70 CG9633-PA	6,30E-13	4,54E-14	2,46
3256111	EG 63B12.10	6,60E-13	4,74E-14	2,46
24647885	14-3-3epsilon CG31196-PA, isoform A	7,09E-13	5,07E-14	2,46
24668824	Mes2 CG11100-PB, isoform B	9,88E-13	7,04E-14	2,44
30059936	nucleoporin 98-96	1,23E-12	8,72E-14	2,43
21358499	CG7430-PA	1,26E-12	8,90E-14	2,43
24643409	CG11943-PB, isoform B	1,50E-12	1,06E-13	2,42
156927	annexin IX	1,64E-12	1,15E-13	2,42
20130301	CG17280-PA	1,70E-12	1,19E-13	2,42
40215478	SD02276p	1,95E-12	1,35E-13	2,41
17738015	Peroxiredoxin 5037 CG5826-PA	2,16E-12	1,50E-13	2,40
16183595	GM01970p	2,77E-12	1,91E-13	2,39
14161109	PDGF/VEGF receptor	3,00E-12	2,06E-13	2,39
21357739	Glycoprotein 93 CG5520-PA	3,27E-12	2,24E-13	2,38
40215636	RE30726p	3,47E-12	2,37E-13	2,38
21357445	CG12360-PA, isoform A	3,69E-12	2,50E-13	2,38
20129901	Ribosomal protein S11 CG8857-PA, isoform A	4,08E-12	2,76E-13	2,37
6694274	ubiquitin-like protein activating enzyme	4,13E-12	2,78E-13	2,37
21357741	CG4951-PA	4,22E-12	2,83E-13	2,37
1770214	smallminded	4,44E-12	2,97E-13	2,37
15292497	SD04165p	4,88E-12	3,25E-13	2,36
290260	phosphoprotein phosphatase 2A 55 kDa regulatory subunit	5,68E-12	3,77E-13	2,36
7109190	gag protein	6,83E-12	4,52E-13	2,35
908756	unknown protein	6,86E-12	4,52E-13	2,35
8886088	eukaryotic initiation factor 5A	7,38E-12	4,85E-13	2,34
24656802	karst CG12008-PA, isoform A	7,93E-12	5,19E-13	2,34
158517	su(s) protein	8,43E-12	5,49E-13	2,34
157365	enhancer of split protein	8,57E-12	5,56E-13	2,34
21356361	Arginine methyltransferase 1 CG6554-PA	8,64E-12	5,59E-13	2,34
24665049	Signal sequence receptor beta CG5474-PA	1,15E-11	7,40E-13	2,32
21357319	Rpn5 CG1100-PA	1,24E-11	7,93E-13	2,32
17647857	Replication-factor-C 40kD subunit CG14999-PA	1,34E-11	8,56E-13	2,32
2597859	u-shaped	1,42E-11	9,03E-13	2,31
24653791	CG8092-PA, isoform A	1,47E-11	9,34E-13	2,31
21357041	CG12171-PA	1,63E-11	1,03E-12	2,31
24645861	Translationally controlled tumor protein	4,00E-11	2,52E-12	2,26

	CG4800-PA			
24662946	Ribosomal protein L10Ab CG7283-PA, isoform A	4,27E-11	2,68E-12	2,26
24641198	GTP-binding protein CG2522-PA	5,23E-11	3,27E-12	2,25
17737663	Rab-protein 8 CG8287-PA	5,24E-11	3,27E-12	2,25
17137738	Rpt1 CG1341-PA	5,62E-11	3,49E-12	2,24
25009816	AT16867p	5,82E-11	3,60E-12	2,24
21357875	CG3731-PB, isoform B	6,70E-11	4,14E-12	2,23
18860521	Ulp1 CG12359-PA	1,05E-10	6,43E-12	2,21
21355085	CG7891-PA	1,21E-10	7,43E-12	2,20
19921528	Asparaginyl-tRNA synthetase CG10687-PA	1,66E-10	1,01E-11	2,19
24581952	Helicase at 25E CG7269-PA, isoform A	1,67E-10	1,02E-11	2,19
287945	ATP synthase beta subunit	1,87E-10	1,13E-11	2,18
19921944	CG1516-PE, isoform E	1,96E-10	1,18E-11	2,18
20428641	26-29kD-proteinase CG8947-PA	2,30E-10	1,38E-11	2,17
21357745	Glutamine fructose-6-phosphate aminotransf 2 CG1345-PA	2,45E-10	1,47E-11	2,17
8170	unnamed protein product	2,90E-10	1,74E-11	2,16
24640873	CG1354-PA, isoform A	4,01E-10	2,39E-11	2,14
21355871	CG1309-PA	4,65E-10	2,77E-11	2,13
62862480	CG40042-PA.3	4,77E-10	2,83E-11	2,13
24644197	CG11999-PA	5,22E-10	3,08E-11	2,13
17137288	Succinyl coenzyme A synthetase flavoprotein subunit CG17246-PA, isoform A	6,44E-10	3,79E-11	2,11
5734514	drosophila dodeca-satellite protein 1	7,53E-10	4,42E-11	2,11
19921254	Ribosomal protein L24 CG9282-PA	7,60E-10	4,44E-11	2,11
24640745	Oligosaccharyltransferase 48kD subunit CG9022-PA	8,69E-10	5,06E-11	2,10
24667974	Z4 CG7752-PA	9,90E-10	5,75E-11	2,09
17944455	RH03140p	1,07E-09	6,20E-11	2,09
158767	ubiquitin	1,16E-09	6,67E-11	2,08
8286	Protein claret segregational	1,51E-09	8,68E-11	2,07
11993642	integral membrane pore glycoprotein gp210	1,62E-09	9,25E-11	2,06
17530825	Ribosomal protein L7A CG3314-PD, isoform D	1,64E-09	9,36E-11	2,06
24582962	CG9541-PA	1,89E-09	1,08E-10	2,06
17137000	Clathrin heavy chain CG9012-PA, isoform A	2,05E-09	1,17E-10	2,05
3056723	translation initiation factor eIF4G	2,28E-09	1,29E-10	2,05
21356113	sec13 CG6773-PA	2,42E-09	1,36E-10	2,04
393376	ETS like protein	2,77E-09	1,56E-10	2,03
18859665	CG11092-PA	3,79E-09	2,12E-10	2,02
21358129	Spt5 CG7626-PA, isoform A	4,54E-09	2,54E-10	2,01
7025386	La related protein	4,75E-09	2,64E-10	2,00
24643725	Helicase CG1666-PA	5,85E-09	3,25E-10	1,99
19921778	spenito CG2910-PB, isoform B	5,90E-09	3,27E-10	1,99
17944889	LD29726p	9,83E-09	5,42E-10	1,96
17530887	IGF-II mRNA-binding protein CG1691-PA, isoform A	1,25E-08	6,85E-10	1,95
18860063	Rpt3 CG16916-PA	1,40E-08	7,66E-10	1,94
19921068	CG5366-PA	1,59E-08	8,70E-10	1,93
28574941	Signal recognition particle receptor beta CG33162-PA	1,66E-08	9,02E-10	1,93

22024280	CG3608-PA	1,89E-08	1,03E-09	1,92
21355167	CG14648-PA, isoform A	2,24E-08	1,21E-09	1,91
18251232	multiple ankyrin repeat single KH domain protein	2,42E-08	1,31E-09	1,91
24655252	Puromycin sensitive aminopeptidase CG1009-PC, isoform C	2,68E-08	1,44E-09	1,90
24657346	CG1295-PA	3,10E-08	1,67E-09	1,89
17864452	Ribosomal protein L12 CG3195-PA, isoform A	3,20E-08	1,71E-09	1,89
21430584	RE12054p	4,26E-08	2,27E-09	1,88
17136866	ADP ribosylation factor 79F CG8385-PB, isoform B	4,72E-08	2,51E-09	1,87
8476	unnamed protein product	6,71E-08	3,56E-09	1,85
17737759	Karyopherin beta 3 CG1059-PA	8,46E-08	4,47E-09	1,83
17136632	porin CG6647-PA, isoform A	9,34E-08	4,92E-09	1,83
17864676	thioredoxin peroxidase 2 CG1274-PA, isoform A	9,57E-08	5,03E-09	1,83
45554154	Vap-33-1 CG5014-PA, isoform A	1,07E-07	5,61E-09	1,82
17864292	Aconitase CG9244-PB	1,16E-07	6,05E-09	1,81
24661707	Ribosomal protein S9 CG3395-PA, isoform A	1,22E-07	6,35E-09	1,81
4580727	phosphate transporter precursor	1,23E-07	6,36E-09	1,81
24653595	Heat shock protein cognate 5 CG8542-PA	1,25E-07	6,49E-09	1,81
4877972	RanGAP	1,48E-07	7,61E-09	1,80
17136620	microtubule star CG7109-PA	1,52E-07	7,81E-09	1,80
18858175	CG7033-PA, isoform A	1,56E-07	7,99E-09	1,80
21356771	zetaCOP CG3948-PA, isoform A	1,59E-07	8,12E-09	1,79
1632782	Fo-ATP synthase subunit b	1,62E-07	8,27E-09	1,79
2795859	MAP kinase	1,81E-07	9,18E-09	1,79
24581914	cyclope CG14028-PA	1,85E-07	9,37E-09	1,78
3337433	transitional endoplasmic reticulum ATPase TER94	2,00E-07	1,01E-08	1,78
24585145	lethal (2) 37Cc CG10691-PA, isoform A	2,60E-07	1,31E-08	1,76
13958630	bicoid mRNA stability factor	3,46E-07	1,74E-08	1,74
24651125	ATP synthase-gamma chain CG7610-PA, isoform A	3,78E-07	1,90E-08	1,74
18860011	CG9911-PA, isoform A	4,59E-07	2,29E-08	1,73
7739653	rasputin	5,86E-07	2,92E-08	1,71
158224	RNA binding protein	6,37E-07	3,16E-08	1,70
6694276	ubiquitin-like protein activating enzyme N subunit	1,29E-06	6,37E-08	1,66
24585152	CG17347-PA	1,54E-06	7,59E-08	1,64
24652825	Megator CG8274-PA	1,66E-06	8,17E-08	1,64
21356141	CG11811-PA	1,86E-06	9,14E-08	1,63
8094	Hrb87F	1,98E-06	9,69E-08	1,63
18079273	stress-sensitive B CG16944-PA, isoform A	2,21E-06	1,08E-07	1,62
24648946	sar1 CG7073-PA, isoform A	2,49E-06	1,21E-07	1,61
20177117	RE37107p	2,87E-06	1,39E-07	1,60
24642361	CG8928-PA	3,46E-06	1,68E-07	1,59
21357545	CG8790-PA, isoform A	3,50E-06	1,69E-07	1,59
24658560	bellwether CG3612-PA	3,64E-06	1,76E-07	1,58
11245459	proteosome subunit RPN2	4,04E-06	1,94E-07	1,58
17864264	Ribosomal protein L30 CG10652-PA, isoform A	4,32E-06	2,07E-07	1,57

21358101	CG1104-PA, isoform A	4,45E-06	2,13E-07	1,57
157658	heat shock protein cognate 72	4,64E-06	2,21E-07	1,57
17136706	Roughened CG1956-PA	5,24E-06	2,49E-07	1,56
16198344	Mud protein	5,71E-06	2,71E-07	1,55
24640442	CG1444-PA	5,84E-06	2,76E-07	1,55
24586067	Eb1 CG3265-PC, isoform C	6,92E-06	3,26E-07	1,54
17136290	Lamin CG6944-PA	7,26E-06	3,42E-07	1,54
45552128	Ranbp16 CG33180-PB	7,70E-06	3,61E-07	1,53
1699220	D-ERp60	1,12E-05	5,25E-07	1,50
19921872	Mystery 45A CG8070-PA	1,54E-05	7,20E-07	1,48
19921016	CG5885-PA	1,91E-05	8,88E-07	1,46
21064855	SD14970p	1,99E-05	9,25E-07	1,46
13124189	Probable elongation factor 1-beta (EF-1-beta)	2,28E-05	1,06E-06	1,45
24647062	Centrosomal protein 190kD CG6384-PA, isoform A	2,71E-05	1,25E-06	1,44
45550174	CG4901-PA	3,19E-05	1,47E-06	1,42
24652542	CG7637-PA	3,58E-05	1,64E-06	1,41
24646105	glorund CG6946-PA, isoform A	3,67E-05	1,68E-06	1,41
1098306	snr1 gene	5,08E-05	2,32E-06	1,39
24580923	CG31938-PA	5,28E-05	2,41E-06	1,38
24648682	Rab-protein 1 CG3320-PA, isoform A	5,55E-05	2,52E-06	1,38
6048198	importin beta	7,01E-05	3,18E-06	1,36
157012	brahma protein	0,00012	5,29E-06	1,32
22024201	CG30122-PB	0,00013	5,75E-06	1,31
5815245	SANT domain protein SMRTER	0,00013	5,81E-06	1,31
28573278	CG18584-PA	0,00018	8,24E-06	1,28
311997	eukaryotic translation initiation factor 4A (eIF-4A)	0,00019	8,59E-06	1,28
17137718	A kinase anchor protein 200 CG13388-PA, isoform A	0,00035	1,58E-05	1,22
13507075	host cell factor HCF	0,00042	1,88E-05	1,21
20129061	CG14224-PA	0,00062	2,77E-05	1,17
27764453	lingerer protein type1	0,00066	2,92E-05	1,17
140834	Retrovirus-related Gag polyprotein from copia-like transposable element 17.6	0,00084	3,71E-05	1,14
21355083	cathD CG1548-PA	0,00144	6,37E-05	1,09
903942	LATS	0,00192	8,45E-05	1,06
158489	alpha-spectrin	0,00201	8,84E-05	1,06
17647799	Protein disulfide isomerase CG6988-PA, isoform A	0,00205	8,96E-05	1,06
19922662	capping protein alpha CG10540-PA	0,00207	9,02E-05	1,05
24650584	without children CG5965-PA	0,00208	9,04E-05	1,05
17136538	Topoisomerase 2 CG10223-PA	0,00217	9,43E-05	1,05
19920620	CG3542-PA, isoform A	0,00244	0,00011	1,04
24641825	CG2691-PA	0,00248	0,00011	1,04
24650838	Heterogeneous nuclear ribonucleoprot at 98DE CG9983-PD	0,00284	0,00012	1,02
158416	sarco/endoplasmic reticulum-type Ca-2+-ATPase	0,00468	0,00020	0,97
20151909	SD03319p	0,00476	0,00020	0,97
17137220	Rab-protein 6 CG6601-PA	0,00654	0,00028	0,93
24651753	fat facets CG1945-PA, isoform A	0,00962	0,00041	0,89
5420159	FL(2)D protein	0,01187	0,00050	0,86

22024213	CG16716-PB, isoform B	0,01514	0,00064	0,83
28557557	SD25413p	0,01797	0,00076	0,81
20129377	CG13393-PA	0,01823	0,00077	0,81
28572980	CG17802-PA	0,02183	0,00091	0,78
21358227	CG1458-PA	0,02416	0,00101	0,77
24652322	14-3-3zeta CG17870-PD, isoform D	0,02662	0,00111	0,76
6006748	hnRNP K protein homolog	0,03348	0,00139	0,73
17738151	Tat-binding protein-1 CG10370-PA	0,03553	0,00147	0,72
24649731	CG5794-PD, isoform D	0,04598	0,00190	0,68

* The table includes interactors that were detected in three independent LC-MS/MS experiments.
