# Supplementary material for: An Interaction between RRP6 and SU(VAR)3-9 Targets RRP6 to Heterochromatin and Contributes to Heterochromatin Maintenance in Drosophila melanogaster
Source: PLoS Genet. 2015 Sep 21;11(9):e1005523. doi: 10.1371/journal.pgen.1005523 (PMC4577213; doi:10.1371/journal.pgen.1005523)
Supplement: S2 Table — (PDF) [file pgen.1005523.s017.pdf]

Table S2. List of the most increased and most decreased transposon insertion sites

INCREASED

Transposon insertion site	Transposon type
Doc3{}571	non_LTR_retrotransposon
Stalker2{}736	LTR_retrotransposon
NOF{}735	DNA transposon/ terminal_inverted_repeat_element
diver{}873	LTR_retrotransposon
blood{}1462	LTR_retrotransposon
G4{}570	non_LTR_retrotransposon
1360{}1481	DNA transposon/ terminal_inverted_repeat_element
1360{}517	DNA transposon/ terminal_inverted_repeat_element
transib3{}567	DNA transposon/ terminal_inverted_repeat_element
diver2{}1137	LTR_retrotransposon
invader5{}566	LTR_retrotransposon
Cr1a{}575	non_LTR_retrotransposon
blood{}356	LTR_retrotransposon
412{}882	LTR_retrotransposon
copia{}1466	LTR_retrotransposon
1360{}569	DNA transposon/ terminal_inverted_repeat_element
Tirant{}1399	LTR_retrotransposon
jockey{}288	non_LTR_retrotransposon
1360{}1138	DNA transposon/ terminal_inverted_repeat_element
aurora-element{}589	LTR_retrotransposon

DECREASED

Transposon insertion site	Transposon type
3S18{}1090	LTR_retrotransposon
412{}904	LTR_retrotransposon
S-element{}1357	DNA transposon/ terminal_inverted_repeat_element
jockey{}307	non_LTR_retrotransposon
pogo{}921	DNA transposon/ terminal_inverted_repeat_element
roo{}952	LTR_retrotransposon
roo{}366	LTR_retrotransposon
roo{}314	LTR_retrotransposon
roo{}1429	LTR_retrotransposon
