# Supplementary material for: An Interaction between RRP6 and SU(VAR)3-9 Targets RRP6 to Heterochromatin and Contributes to Heterochromatin Maintenance in Drosophila melanogaster
Source: PLoS Genet. 2015 Sep 21;11(9):e1005523. doi: 10.1371/journal.pgen.1005523 (PMC4577213; doi:10.1371/journal.pgen.1005523)
Supplement: S3 Table — (PDF) [file pgen.1005523.s018.pdf]

Table S3. RRP6-bound genes showing differential transcript levels (log2 >1) in RRP6-depleted cells

INCREASED

CG31279	CG32111	CG34155	CG4467	Scp2	fz2
CR43857	CG34172	CG34114	acj6	CG2254	peb
CG14692	CR43610	E23	CG31004	CG43902	pog
os	side	verm	CG32698	AlstR	CG11966
salr	CG12541	CG17764	CG3921	CG2901	m
CG10625	CG14326	Ir40a	cas	CG42749	CG15753
CG13506	CG42329	W	Lkr	CG14837	ckd
Nep3	CG9400	ham	CG42708	CG4168	w
Dscam2	dpr8	dan	CG41520	CG42594	DAT
abba	CG17684	gk	nop5	elk	para
CG11693	Rim	sdt	CR43801	CG9782	Side
CG43265	kirre	Gk	CG30110	dally	CG6928
CG8065	cato	CG4404	Nckx30C	TrxT	CG17839
CG9095	CG14010	phm	CG43347	tutl	Pop2
Snap25	lobo	Cht7	CG43796	Cpr76Bd	Socs36E
upd3	pdm2	Hdc	D	dnr1	CG42342
CG7884	CG32720	D2R	dpr	CG34380	GC
qvr	CG31475	CG31191	Rdl	sano	mmd
CR43461	CR43334	Fas3	Sulf1	CG11317	gl
CG34347	CG2256	mun	CG6520	CG34354	CG1443
bi	Sox100B	Fas2	otp	CG34391	CG33988
CG14521	nkd	CG7691	Cng	CG7330	CG12531
SIP2	CG13743	CG13838	CG43462	CR43960	CG3168
CG2022	CG43109	CG43290	CG30419	nvd	ASPP
CG14661	Gef64C	bab2	stan	CG3198	CG34353
CR43283	Mocs1	gprs	Fhos	CG33143	mfr
dys	CG31808	CG18675	Clk	TyrRII	sn
CG42683	CR34052	hbs	Eip93F	CG13954	Btd
CR43650	vn	Sur	MRP	Spargel	futsch
eya	Nos	Pkcdelta	CG14324	CG42524	sca
CG15572	sr	CG31626	CG18302	Lasp	d
Dr	CG34386	fas	CG42534	Rgk1	Ast
CG42673	CG34286	Teh2	CG8861	CG4374	CG42613
CR43951	CG9650	Sobp	CR41621	CG33158	trx
Syt12	Pdfr	Glut1	Frq2	ogre	CG9287
CG42732	Fili	sns	Mip	Dip2	
CG15765	CG34113	CG13837	RpS28a	ptr	

DECREASED

Pkc53E	CG14185	LRP1	CG43797	Cyp4g15	Cpr65Eb
CG14180	CG12061	CG17341	CG31663	pcl	Vsx2
CR40546	CG42343	CG7224	CG10361	CG2111	
vkg	CG40467	retn	klg	btd	
CG1092	Ubx	Dl	CG14292	CG32105	
Trh	CG10249	SK	CG43676	Trim9	
