# Supplementary material for: An Interaction between RRP6 and SU(VAR)3-9 Targets RRP6 to Heterochromatin and Contributes to Heterochromatin Maintenance in Drosophila melanogaster
Source: PLoS Genet. 2015 Sep 21;11(9):e1005523. doi: 10.1371/journal.pgen.1005523 (PMC4577213; doi:10.1371/journal.pgen.1005523)
Supplement: S4 Table — (PDF) [file pgen.1005523.s019.pdf]

Table S4. Effect of RRP6 depletion on the expression of RRP6-bound genes

	Number of genes in each class	Average expression (RNA-seq signal in GFP_control)	Nr. genes with increased expression in RRP6_kd (log2 ratio > 1)	Average fold change RRP6_kd vs GFP_control (log2 ratio > 1)
All genes	13272	1,699	1534	0,409
RRP6-bound	967	2,666	220	0,421
SUV-dependent	321	3,169	63	0,437
SUV-independent	646	2,416	142	0,412
