# Supplementary material for: An Interaction between RRP6 and SU(VAR)3-9 Targets RRP6 to Heterochromatin and Contributes to Heterochromatin Maintenance in Drosophila melanogaster
Source: PLoS Genet. 2015 Sep 21;11(9):e1005523. doi: 10.1371/journal.pgen.1005523 (PMC4577213; doi:10.1371/journal.pgen.1005523)
Supplement: S1 Text — (DOCX) [file pgen.1005523.s020.docx]

Eberle et al.

**Text S1: Supplementary Materials and Methods**

***Plasmid preparation***

The open reading frame of SU(VAR)3-9 was amplified by PCR using a forward primer with a *Spe*I restriction site (5’-AATT*ACTAGT***ATG**GCCACGGCTGAAGCC-3’) and a reverse primer containing an HAtag, a **stop codon** and a *Xho*I restriction site (5’- TAAA*CTCGAG***TCA**AGCGTAATCTGGAACATCGTATGGGTAAAAGAGGACCTTTCTGCAATTATC-3’). Since the PCR fragment was too long to amplify successfully, two overlapping PCR products were generated with an overlapping *Apa*I restriction site for the ligation of the two intermediate fragments (forward primer + 5’-GTGACGGTTTCTCCACATGA-3’ for first intermediate amplicon and 5’-ACGCCTATGAGCCACTCAAT-3’ and reverse primer for second intermediate amplicon). The assembled PCR product was next digested with *Spe*I and *Xho*I and ligated with the digested pMT-puro Drosophila expression vector (addgene). The ligation product was transformed into *E. coli* XL1 gold cells and the obtained plasmid confirmed by restriction analysis and sequencing.

***Immunoprecipitation and high-performance liquid chromatography/tandem mass spectrometry***

Immunoprecipitation and high-throughput mass spectrometry were performed by Hessle et al. [34] using nuclear protein extracts prepared from S2 cells that expressed V5-tagged RRP6. An “empty” control cell line that was cultivated under the same conditions but did not express any V5-tagged protein was processed in parallel. The protein extracts used for the mass spectrometry studies were nuclear protein extracts digested with RNase A. Three independent immunoprecipitation experiments followed by HPLC/MS/MS analysis were carried out, and each experiment included an immunoprecipitation with the “empty” cells processed in parallel. Protein abundances were normalized by the abundance of the immunoglobulin heavy-chain variable region [Mus musculus] (gi 27728890). Significant interactions were identified using a false discovery rate (FDR) threshold of 0.01, as well as a minimum fold-difference of 2 compared to “empty” cells. The complete list of interactors (including interactors detected in all three experiments) is provided as Suppl. Table S1. Functional annotation and GO enrichment analysis was performed by DAVID [49] with the parameter Ease=0.01.

***SDS-PAGE and Western Blotting***

SDS-PAGE and Western blotting were performed according to standard procedures (see also reference [50]). Image Lab 3.0 Software from BioRad was used to quantify Western blots.

***RNA interference in S2 cells***

Long double-stranded RNAs (dsRNAs) against GFP (control), RRP6, DIS3, HP1a and SU(VAR)3-9 were prepared by in vitro transcription of PCR products with T7 promoters on both ends using the Megascript RNAi kit (Ambion; oligos are listed in the table below).

Three millions S2 cells were seeded in a 35mm dish the day before the first transfection of 20 µg dsRNA. After 48 hours, the cells were treated again with 20 µg dsRNA, and after 48 hours the cells were harvested.

***RNA-seq***

S2 cells were treated with dsRNA to knock down RRP6. Control cells were treated in parallel with dsRNA that was complementary to GFP. Total RNA was purified using the RNAqueous kit (Ambion) after four days of dsRNA treatment, as indicated above. The RNA was ribosome depleted using the Ribo-Zero rRNA Removal Kit (Illumina) and used to construct random-primed cDNA libraries for next-generation sequencing. The libraries were prepared and sequenced by GATC-Biotech AB on a HiSeq2500 sequencer to a depth of at least 30 M single-end reads per sample. RNA samples from two independent experiments were sequenced in parallel, each with a GFP control sample. Illumina fastq files where inspected with FastQC to assess quality of the reads. High quality reads were mapped with TopHat2 [52] to the *Drosophila melanogaster* genome assembly, build BDGP6 (Dm3). BAM alignments were indexed with SAMtools (Li et al, 2009) and visualized in IGV browser (https://www.broadinstitute.org/igv/) [53]. Read counts per gene were generated using HTSeq (Anders et al, 2014) on bam files. Mapping statistics were calculated from numbers obtained by running the phython script read_distribution.py included in the RSeQC package (Wang et al, 2012) on bam files. Differential expression analysis was carried out using Limma package in R (Ritchie et al, 2015). The data generated in this study are available at NCBI’s Gene Expression Omnibus (accession number GSE66640).

***Micrococcal nuclease (MNase) experiment***

Drosophila cells were crosslinked at room temperature for 10 min with 1 % formaldehyde. After incubation with 0.1 M glycine for 10 min, the cells were centrifuged at 500 g for 5 min. The pellet was resuspended in cold buffer 1 (50 mM Hepes pH 7.6, 140 mM NaCl, 1 mM EDTA, 10 % glycerol, 0.5 % Nonident P40, 0.2 % Triton-X, complete protease inhibitor (Roche)) and incubated for 10 min at 4 °C. After a second centrifugation, the pellet was dissolved in buffer 2 (10 mM Tris pH 8, 200 mM NaCl, 1 mM EDTA, 0.5 mM EGTA, protease inhibitors) and incubated again for 10 min at 4 °C. After the next centrifugation, the pellet was resuspended in buffer D (25 % glycerol, 5 mM Magnesium acetate, 50 mM Tris pH8, 0.1 mM EDTA and 5 mM DTT). After an incubation of 10 min at 4 °C, the sample was spun down and the obtained pellet dissolved in MNase buffer (60 mM KCl, 15 mM NaCl, 15 mM Tris pH 7.5, 0.5 mM DTT, 0.25 M sucrose and 1.0 mM CaCl_2_). After a very mild sonication, 20 U micrococcal nuclease (MNase; New England Biolabs) was added and incubated for 30 min at 37 °C. The reaction was stopped by the addition of 12.5 mM EDTA and 0.5 % SDS. Next, proteins were degraded by proteinase K and the crosslinking was reversed over night at 65 °C. DNA was extracted with phenol:chloroform and precipitated with ethanol in the present of salt and glycogen. The amount of DNA was measure by qPCR and the data was normalized to a nucleosome free region in the heat shock protein Hsp70 [39].

***Immunofluorescent staining of polytene chromosome squashes***

Salivary glands were dissected from third instar larvae, fixed in a solution containing 3.7% formaldehyde and 45% acetic acid in PBS for 10 minutes, and squashed in a drop of 45% acetic acid. The slides were frozen on dry ice, the coverslips pried off, and the preparations were further fixed with 3.7% formaldehyde in PBS for 15 min. The slides were washed with PBS containing 0.1 M glycine, and blocked with a solution of 3% BSA in PBSG. The slides were incubated with anti-RRP6 antibody [20] and anti-HP1a antibody. The chromosomes were washed, incubated with fluorescent secondary antibodies, mounted with Vectashield mounting medium (Vector Laboratories Inc.) and examined in an LSM 510 laser confocal microscope (Carl Zeiss). The optical sections are approximately 1 µm thick. Co-localization was analyzed using the *Profile* function of the LSM 510 software by drawing a test line along the region of interest and measuring the relative fluorescence intensity along the line in arbitrary units.

***Immunofluorescent staining of S2 cells***

S2 cells were allowed to adhere to glass slides coated with poly-L-lysine in a humid chamber and fixed in 3.7% PBS for 10 min at room temperature. The fixed cells were permeabilized in 0.1 % Triton-X100 in PBS for 13 min at room temperature. The cells were blocked in 5% milk and 5% BSA in PBS and incubated with primary antibodies following standard procedures. After the antibody incubations, the slides were mounted with Vectashield mounting medium (Vector Laboratories Inc.) and examined in either an Axioplan fluorescence microscope (Carl Zeiss) or an LSM 510 laser confocal microscope (Carl Zeiss). The optical sections were approximately 0.8 µm thick.

***Immuno-electron microscopy***

S2 cells were fixed for 45 min in 4% paraformaldehyde, cryoprotected with 2.3 M sucrose, frozen by immersion in liquid nitrogen and cryosectioned. Thin cryosections were picked up on drops of 2.3 M sucrose and mounted onto nickel grids coated with formvar and carbon. The grids were floated onto drops of PBS containing 0.1 M glycine and 10% fetal calf serum before incubation with the antibody solutions. The primary antibodies were anti-RRP6 antibody and anti-HP1a antibody diluted in PBS containing 0.05 M glycine and 5 % fetal calf serum. The secondary antibodies were conjugated to 6 nm and 12 nm gold particles (Jackson ImmunoResearch Laboratories). After immunolabelling, the sections were stained with 2% aqueous uranyl acetate for 3-5 min and embedded in polyvinyl alcohol (9-10 kDa, Aldrich). The specimens were examined and photographed in a FEI Tecnai G2 electron microscope at 80 kV.

***Co-immunoprecipitation experiments***

S2 cells were washed once in PBS, resuspended in lysis buffer (PBS with 0.2% Nonident P40 and Complete protease inhibitor (Roche)) and homogenized using a glass homogenizer with a tight pestle B. After centrifugation at 2000g for 10 min at 4 °C, the supernatant (corresponding to the cytoplasm) was discarded. The pellet was resuspended in PBS that contained complete protease inhibitor, sonicated four times for 3-4 pulses each, and centrifuged at 16,000g for 15 min at 4 °C. The supernatant was used for co-immunoprecipitation with the corresponding antibody. After one to two hours of incubation, a 1:1 mixture of Sepharose-Protein A and Sepharose-Protein G beads (Invitrogen and GE Healthcare) was added to the immunoprecipitation reaction and the incubation was allowed to proceed for one additional hour. The samples were washed five times with PBS containing 0.1% Nonident P40 and complete protease inhibitor and eluted with 1% SDS at room temperature. The samples were analysed by SDS-PAGE and Western blotting.

***Fractionation of the cell nucleus***

S2 cells were resuspended in cold lysis buffer (PBS containing 0.2% Nonident P40 substitute and Complete protease inhibitor (Roche)) and homogenized in a glass homogenizer with a tight pestle B. The homogenized sample was centrifuged at 1500g for 10 min at 4 °C. The pellet (nuclei) was dissolved in PBS that contained complete protease inhibitor, sonicated three times 3-4 pulses each and centrifuged again at 16,000g for 10 min at 4 °C. The proteins of the supernatant (*soluble* nuclear fraction) were precipitated in acetone. The pellet was resuspended in PBS, incubated with 30 mg RNase A for 15 min at room temperature, and centrifuged again as above. After centrifugation, the proteins of the supernatant (*chromosomal RNP*) were precipitated in acetone and the pellet (*chromatin*) was dissolved in SDS sample buffer. The samples were analysed by SDS-PAGE and Western blotting.

***RNA immunoprecipitation (RIP) experiment***

S2 cells were fixed at room temperature with 1% formaldehyde for 10 min and the chromatin was harvested as for ChIP experiments. After sonication of chromatin, the genomic DNA was degraded for 30 min at 37 °C with 100 U DNases (Thermo Scientific). After addition of EDTA, the samples were spun down at 16,000g for 30 min at 4 °C. Next, the samples were pre-cleared with Sepharose A/G beads (50:50; Invitrogen, GE Healthcare) for 2 hours on a rotating wheel. The same amount of the RNA-protein mixture (measured by OD at 280 nm) was used for all samples. The antibody was incubated overnight at 4 °C in 0.1% deoxycholic acid and 1% Triton X-100. The next day, blocked Sepharose A/G beads were added for one hour. The samples were then washed five times with RIPA buffer (50 mM Hepes at pH 7.6, 500 mM NaCl, 1 mM EDTA, 1% Nonident P40, 0.8% deoxycholic acid) and once with 50 mM Tris at pH 8 and 2 mM EDTA. The pellet was incubated in TE-buffer that included SDS, proteinase K and RNaseOUT for 30 min at 42 °C and then moved to 65 °C for one hour to reverse the crosslinking. RNA was extracted with Trizol (Invitrogen) and the total RNA was transcribed into cDNA and analyzed with qPCR.

***cDNA preparation***

RNA from S2 cells was isolated using the RNAqueous kit (Ambion). The RNA was treated with DNAse (Thermo Scientific) for 30 min at 37 °C to remove any residual DNA contamination. Reverse transcription was performed with Superscript-III (Invitrogen) using random hexamers (Invitrogen). RT (–) controls were carried out to monitor DNA contamination.

***ChIP-seq***

RRP6-V5 cells treated with dsRNA for either GFP (controls) or Su(Var)3-9 were used for ChIP-seq with anti-V5 antibody. ChIP was performed as described in [54]. DNA was purified from input and immunoprecipitated chromatin. Material from three independent RNAi-ChIP experiments was pooled and sequenced. DNA libraries were prepared by Zymo Research Epigenetics Services and were sequenced on a HiSeq2500 sequencer. RRP6 ChIP-Seq reads were aligned to the *D. melanogaster* reference genome dm3 by Bowtie with at most 2 mismatches. Reads that appeared more than twice at the same position on the same strand were discarded to remove PCR duplication. BIGWIG files were generated from the coverage for visualization purposes. MACS2 v2.1.0.20140616 was used to identify RRP6-rich regions at q-value cutoff 0.01 using broad peak setting. In addition, RRP6-rich regions overlapping by less than 50 % in GFP controls and Su(Var)3-9-KD were considered ‘‘common’’ to both samples. Otherwise the two regions were considered ‘‘unique’’.

***References not included in the main text:***

Anders, S., Pyl, P. T., Huber, W. (2014). HTSeq — A Python framework to work with high-throughput sequencing data. Bioinformatics (2014), in print, online at doi:10.1093/bioinformatics/btu638

Li, H., Handsaker, B., Wysoker, A., Fennell, T., Ruan, J., et al. (2009). The Sequence alignment/map (SAM) format and SAMtools. Bioinformatics, 25, 2078-2079.

Ritchie, M.E., Phipson, B., Wu, D., Hu, Y., Law, C.W. et al. (2015). Limma powers differential expression analyses for RNA-sequencing and microarray studies. Nucleic Acids Research, 43(7), pp. e47.

Wang, L., Wang, S., Li, W. (2012) RSeQC: quality control of RNA-seq experiments. Bioinformatics 28, 2184-2185.

***Oligos used in this study***

| Oligo name | Sequences 5’ - 3’ | Used for |
| --- | --- | --- |
| **RNA interference:** |  |  |
| GFP_T7 F | taatacgactcactatagggagaatggtgagcaagggcgaggagctg | dsRNA against GFP (control) |
| GFP_T7 R | taatacgactcactatagggagagcggtcacgaactccagcag | “ “ |
| RRP6_T7 F | taatacgactcactatagggagatctgttgatagacactgccc | dsRNA against RRP6 |
| RRP6_T7 R | taatacgactcactatagggagaattctcttaaaccgctggcc | “ “ |
| DIS3_T7 F | taatacgactcactatagggagagacggaaatggcgacagtat | dsRNA against DIS3 |
| DIS3_T7 R | taatacgactcactatagggagatgatgacgctcttgtggaag | “ “ |
| HP1_T7 F | taatacgactcactatagggagaccctctggcaataaatcaaaa | dsRNA against HP1a |
| HP1_T7 R | taatacgactcactatagggagattaatcttcattatcagagtacca | “ “ |
| SU(VAR)3-9_T7 F | taatacgactcactatagggagaggagaacgagccctgaaaagc | dsRNA against SU(VAR)3-9 |
| SU(VAR)3-9_T7 R | taatacgactcactatagggagacacccttacgcaaggcagttg | “ “ |
| **KD efficiencies:** |  |  |
| HP1a F | agaaaatcgacaaccctgaga | HP1a RNA level |
| HP1a R | gcctgtcgatgatcttttcc | “ “ |
| SU(VAR)3-9 F | cgttcttgaagcgcacagt | SU(VAR)3-9 RNA level |
| SU(VAR)3-9 R | gcctctatcaccggaggtc | “ “ |
| Dis3 F | aaccgtgactggatcaaagg | Dis3 RNA level |
| Dis3 R | gctctgcaagtgctaatccc | “ “ |
| **Heterochromatin analysis:** |  |  |
| Inv1 F | gcttatggccaaatggaaga | Invader forward |
| Inv1 R | gaaaactaaacgccgcaaac | Invader reverse |
| Max F | cattggaagccatcgaagat | Max forward |
| Max R | acaccctttctgtttggtgc | Max reverse |
| LC repeats F | catctcaattgcgctgaaga | Low-compl. repeats forward |
| LC repeats R | tgccttcattcgaaggagtt | Low-compl. repeats reverse |
| Doc F (ae226) | ggccaaaaacttttctgcaa | Doc forward |
| Doc R (ae227) | tagtgcctggagctctggtt | Doc reverse |
| Roo F (ae228) | ggcgttgtcgatcatgaata | Roo forward |
| Roo R (ae229) | accgactcgcccttaatttt | Roo reverse |
| EC repeats F (ae216) | gcctctcattttctctcccata | EC repeats forward |
| EC repeats R (ae217) | gttcatccgccaccatttt | EC repeats reverse |
| Inv4 repeats F (ae224) | cgaaaacttacaaacaaattattgaa | Inv4 repeats forward |
| Inv4 repeats R (ae225) | ccaaaaacagagaacattaaaaa | Inv4 repeats reverse |

| **MNase experiment:** |  |  |
| --- | --- | --- |
| Hsp70 promoter F | ttgtgactctccctctttgt | Hsp70, promoter |
| Hsp70 promoter R | ttcgcgaacattcgaggcg | Hsp70, promoter |
| Hsp70 coding F | attatcgccaacgaccagggcaa | Hsp70, coding region |
| Hsp70 coding R | ttcatggccacctggttctt | Hsp70, coding region |
| **Actin 5C (CG4027)** | **Used for ChIP-qPCR** |  |
| Act 1F | TGT GGG CGT GTC ACG ATT T |  |
| Act 1R | TTT AGG GCG TGG GAA AAA AA |  |
| **Actin 5C (CG4027)** | **Used for normalization** |  |
| Act 2F | gcacacccacaagcttacaca |  |
| Act 2R | ttgcgctttgggaaatatcttc |  |
| **PGK (CG3127)** |  |  |
| Pgk F (ae94) | GTGGAACGGACCCCCC | Pgk forward |
| Pgk R (ae157) | GAGACGGTGCCGTTCTTG | Pgk reverse |
